# Genome-wide identification and characterization of mRNAs and lncRNAs involved in cold stress in the wild banana (*Musa itinerans*)

**DOI:** 10.1371/journal.pone.0200002

**Published:** 2018-07-09

**Authors:** Weihua Liu, Chunzhen Cheng, Yuling Lin, Xu XuHan, Zhongxiong Lai

**Affiliations:** 1 Institute of Horticultural Biotechnology, Fujian Agriculture and Forestry University, Fuzhou, Fujian, China; 2 Institut de la Recherche Interdisciplinaire de Toulouse, IRIT-ARI, Toulouse, France; Bhabha Atomic Research Centre, INDIA

## Abstract

Cold stress seriously affects banana growth, yield and fruit quality. Long noncoding RNAs (lncRNAs) have been demonstrated as key regulators of biotic and abiotic stress in plants, but the identification and prediction of cold responsive mRNAs and lncRNAs in wild banana remains unexplored. In present study, a cold resistant wild banana line from China was used to profile the cold-responsive mRNAs and lncRNAs by RNA-seq under cold stress conditions, i.e. 13°C (critical growth temperature), 4°C (chilling temperature), 0°C (freezing temperature) and normal growing condition, i.e. 28°C (control group). A total of 12,462 lncRNAs were identified in cold-stressed wild banana. In mRNA, much more alternative splicing events occurred in wild banana under the cold stress conditions compared with that in the normal growing condition. The GO analysis of differential expression genes (DEGs) showed the biochemical processes and membrane related genes responded positively to the cold stress. The KEGG pathway enrichment analysis of the DEGs showed that the pathways of photosynthesis, photosynthesis–antenna proteins, circadian rhythm–plant, glutathione metabolism, starch and sucrose metabolism, cutin/suberine/biosynthesis were altered or affected by the cold stress conditions. Our analyses of the generated transcriptome and lncRNAs provide new insights into regulating expression of genes and lncRNAs that respond to cold stress in the wild banana.

## 1 Introduction

Banana is an economically important crop in tropical and subtropical regions worldwide. Banana is thermophilic, and the normal growth temperature ranges from 15°C to 35°C. The growth low critical temperature of cultivated banana is species-dependent and ranges from 10 to 17°C [1–3], which is about 13°C for most cultivated banana in China [4]. Most of cultivated banana in Fujian province, China were distributed in southern subtropical regions, which are the northern margin of world banana cultivation, the cultivated banana here often suffered the cold stress in winter and early spring. To date, however, effective methods to solve banana cold stress have not been satisfactory, and the banana responses to cold stress have not been investigated by means of high-throughput transcriptome sequencing and long non-coding RNA (lncRNA) analytical methods.

The wild banana is more tolerant to biotic and abiotic stresses compared with the cultivated lines, especially to cold stress [5–6]. China is rich in wild banana genetic resources. A cold tolerant germplasm found from wild banana plants in Sanming city, Fujian province [7–9] is extremely cold resistant, and grows well around 0°C [8]. The semilethal temperature of the wild banana reached as low as -1.776°C, thus, lower than other screened banana resources [9]. Field tests indicated the wild banana was more cold resistant than a cultivated banana ‘Tianbaojiao’, e.g. at 4°C, which was the chilling temperature to the cultivated banana, the leaves of ‘Tianbaojiao’ were wilting, but the wild banana kept normal in morphology even when the temperature went as low as to 0°C (S1 Fig). The wild banana was used for high-throughput analysis in the present study.

Transcriptomics techniques have been applied to investigate the cold-responsive mechanism in many crops [10–16], but *Musa* species were seldom involved. Yang *et al* (2005) conducted a comparative transcriptomic study to identify the key expression-level differences between banana (AAA group, cold-sensitive) and plantain (cold-tolerant) to low temperatures [17]. The results indicated that the majority of the DEGs identified in banana and plantain belonged to 11 categories (e.g. regulation of transcription and response to stress signal transduction), and the banana and the plantain share some common adaptive processes in response to cold stress. Additionally, 17 DEGs specific to cold tolerant plantain were grouped into specific categories, including signal transduction, abiotic stress, copper ion equilibrium, photosynthesis and photorespiration, sugar accumulation and protein modifications, and *ICE1* (*inducer of CBF expression 1*) and *MYBS3* (*avian myeloblastosis viral oncogene homolog S3*) were fond rapidly and pacifistically activated in the cold tolerance pathways of the plantain. These results were considered to explain why plantain was more cold-tolerant than the cultivated banana. However, as to the wild banana species, which may respond to cold stress differently than either the cultivated banana or the plantain species, remains unknown.

Long non-coding RNAs have recently been identified in model plants species [18–20] and in some crops [21–25]. The importance of lncRNAs for plant responses to biotic [26–28] and abiotic stresses [29–31], including cold stress [32], have recently been confirmed. For example, Muthusamy *et al* (2015) [33] conducted a genome-wide screen by an Illumina high-throughput sequencing technique to detect novel, drought responsive lncRNAs in the transcriptomes of drought-stressed leaves from drought-tolerant and -susceptible banana cultivars (*Musa* spp.). In 905 novel lncRNAs identified in a drought-tolerant cultivar ‘Saba’ (ABB group) and a drought-susceptible cultivar, ‘Grand Naine’ (AAA group), 75 (8.3%) transcripts encoded natural antisense RNAs (NATs) and two transcripts encoded precursors of the microRNAs-miR156 and miR166. Among 882 drought-responsive lncRNAs, 44 new lncRNAs were identified as the induced. Approximately 7.9% of the identified lncRNAs were decoys of 85 conserved microRNAs. In general, lncRNAs are poorly conserved among plant species [24, 28, 34].

Physiological and proteomic responses to cold stress are observed in banana [35–37]. Clarifying the cold-induced genome-wide gene expression patterns and changes in lncRNAs levels *via* high throughout RNA-sequencing is needed for elucidating the regulatory pathways mediating cold responses in wild banana. Identifying these regulatory mRNAs and lncRNAs responsive to cold stress will improve our understanding of cold-resistance in banana species and is valuable to cold-tolerance breeding.

## 2 Materials and methods

### 2.1 Plant materials and cold stress treatments

The wild banana (*Musa itinerans*) plants from Sanming city of China, which was preserved in the wild Banana Germplasm Bank of the Institute of Horticultural Biotechnology of Fujian Agriculture and Forestry University, were used in the present study. *In-vitro* plantlets produced by tissue culture from the explants of suckers were transplanted to pots and cultivated for 1 month at 28°C under a 12-h light (fluorescent light, 2000 lx) 12-h dark cycle. Uniformly growing seedlings were selected for the following treatments. Seedlings that had been watered sufficiently for 2 days were placed in 4 growth chambers set at 28°C (control), 13°C, 4°C, or 0°C. In each chamber, 10 seedlings were incubated for 24 h under 12-h light (fluorescent light, 2000 lx) 12-h dark cycle (synchronized with the natural light cycle), with a relative humidity of 70–80%. The first young leaf at the top of each seedling was detached and pooled for each temperature treatment. There biological replicates, with leaves from 10 seedlings per replicate were analyzed for each treatment. The leaf samples were frozen in liquid N_2_ and stored at -80°C for the subsequent extraction of total RNA and analyses of mRNAs and lncRNAs by sequencing and a quantitative real-time polymerase chain reaction (qPCR) assay.

### 2.2 The total RNA extraction

The total RNA was extracted from the wild banana leaves according to the method by Liu *et al* (2015) [7]. The total RNA integrity was qualified, and the RIN values of the four libraries from the treatments at 0°C, 4°C, 13°C, 28°C were 7.8, 7.7, 7.8, 8.1, respectively. The total RNA was used for further sequencing and qPCR analysis.

### 2.3 Library construction and high-throughput sequencing

We constructed cDNA libraries for the leaf samples collected at each temperature treatment (28, 13, 4, 0°C) using the TruSeq Stranded Total RNA Library Prep Kit with Ribo-Zero Gold (Illumina, San Diego, CA, USA) to eliminate rRNA by Novogene (China). The cDNAs were sequenced by Illumina HiSeqTM 4000 at Beijing Novogene Bioinformatics Technology Co., Ltd.

### 2.4 Bioinformatics identification of the wild banana mRNAs and lncRNAs

The computational pipeline for the mRNAs and lncRNAs identification of the wild banana was shown in Panel A in S2 Fig. After removing the low quality (N% > 10%), adapter sequences and other low-quality reads from the sequenced raw data, the clean reads of the four libraries were obtained for further analyses. The clean reads were aligned to the banana reference genome (*Musa accuminata*) by Tophat 2 [38] for mRNA analysis. Novel transcript assembly was conducted by Cufflinks [39]. The number of fragments per kilobase per million mapped reads (FPKM) per gene was calculated [40].

The bioinformatic identifcation of wild banana lncRNAs was presented in Panel B in S2 Fig. The mapped reads of each sample were assembled by Cufflinks [39]. And then, 5 steps strict screening conditions were set up according to the structure characteristics of lncRNA: i) Nearest distance ≥ 500 bp with other transcripts for single-exon transcript; ii) Transcript length ≥ 200; iii) FPKM ≥ 0.5 for multi-exon transcript, FPKM ≥ 2 for single-exon transcript; iv) Filter known non-lncRNA annotation; v) Classification of candidate lncRNAs. In addition, CPC (Coding Potential Calculator; 0.9-r2. The score of noncoding<0) [41] and Pfam database (v1.3, release 27; used both Pfam A and Pfam B; the threshold value is Evalue < 0.001, as non-coding) [42] were used for coding potential filtering according to the function characteristics (non-coding transcripts) of lncRNA. The coding potential and encoding any conserved protein domains of the remaining transcripts were evaluated by CPC and Pfam respectively. Based on all of the above filtered steps, the remained transcripts were considered as candidate lncRNAs for further analysis.

The *cis*-acting targets for lncRNAs were predicted, and coding genes 10k/100k upstream and downstream of lncRNA were searched and their functions were analyzed.

Cuffdiff was used to provide statistical routines for determining differential expression in digital transcript or gene expression data using a model based on the negative binomial distribution [40]. P-adjust < 0.05 and the absolute value of log2 (Fold change) < 1 were set as the threshold of the significantly differential expression.

The differentially expressed (DE) mRNAs were used for GO and KEGG enrichment analysis by GOseq R package [43] and KOBAS software [44–45]. DE lncRNAs and mRNAs were analyzed using the Cuffdiff algorithm. The targets for the DE lncRNAs underwent Gene Ontology (GO) and Kyoto Encyclopedia of Genes and Genomes (KEGG) enrichment analyses with the GOseq R package and KOBAS software. The KOBAS software was used for testing the enrichment of KEGG pathways among the DE lncRNA target genes (http://www.genome.ad.jp/kegg).

### 2.5 qPCR validation of DE mRNAs, lncRNAs and their targets

The extracted total RNAs was used to validate the mRNAs as well as lncRNAs and their targets by qPCR. The total RNAs were reverse transcribed into cDNA with PrimeScript™ RT reagent kit (Prefect Real Time) (Takara, Japan) according to manufacturers’ instructions. Three biological replicates and three technical replicates were assayed. The qPCR was completed by a LightCycler 480 (Roche) as described by Liu *et al* [7], and the expression levels were quantified according to the 2^-ΔΔ^Ct method [7]. Primers were designed using the Primer 3 (http://bioinfo.ut.ee/primer3-04.0/) soft-ware, and are listed in S1 Table. Significant differences in the data were assessed with SPSS, and *P* < 0.01 was considered significant.

## 3 Results

### 3.1 Identification of cold-responsive differential expression genes (DEGs) during cold stress in wild banana

#### 3.1.1 Illumina sequencing, quality control, and DEGs identification of the wild banana transcriptome

To identify DEGs associated with wild banana responses to cold stress, the four cDNA libraries corresponding to the four tested temperatures [i.e., 28°C (control), 13°C (critical growth temperature), 4°C (chilling temperature), 0°C (freezing temperature)] were constructed. The cDNAs were sequenced by Illumina HiSeqTM 4000 at Beijing Novogene Bioinformatics Technology Co., Ltd. The removal of low-quality reads (e.g. N% > 10%) and adapter sequences from the raw data, resulted in 89,782,494 (0°C), 85,862,054 (4°C), 91,566,738 (13°C) and 84,432,094 (28°C) of clean reads, comprising 13.47, 12.88, 13.74 and 12.66 Gb of nucleotide data, respectively (S2 Table).

The Tophats program was used to map clean reads to the banana reference genome. Additionally, Cufflinks was used to assemble novel transcripts. The FPKM distribution and density distribution are presented in S3 Fig. All the FPKM values were > 1, implying the RNA-seq data were normal. The FPKM values for the mRNA from all samples are listed in S3 Table. The DEGs were defined as the genes with an expression level fold-change ≥2 and a false discovery rate (FDR) < 0.05 (S4 and S5 Tables).

#### 3.1.2 The GO and KEGG enrichment analysis of DEGs responsive to cold stress

**Fig 1:**
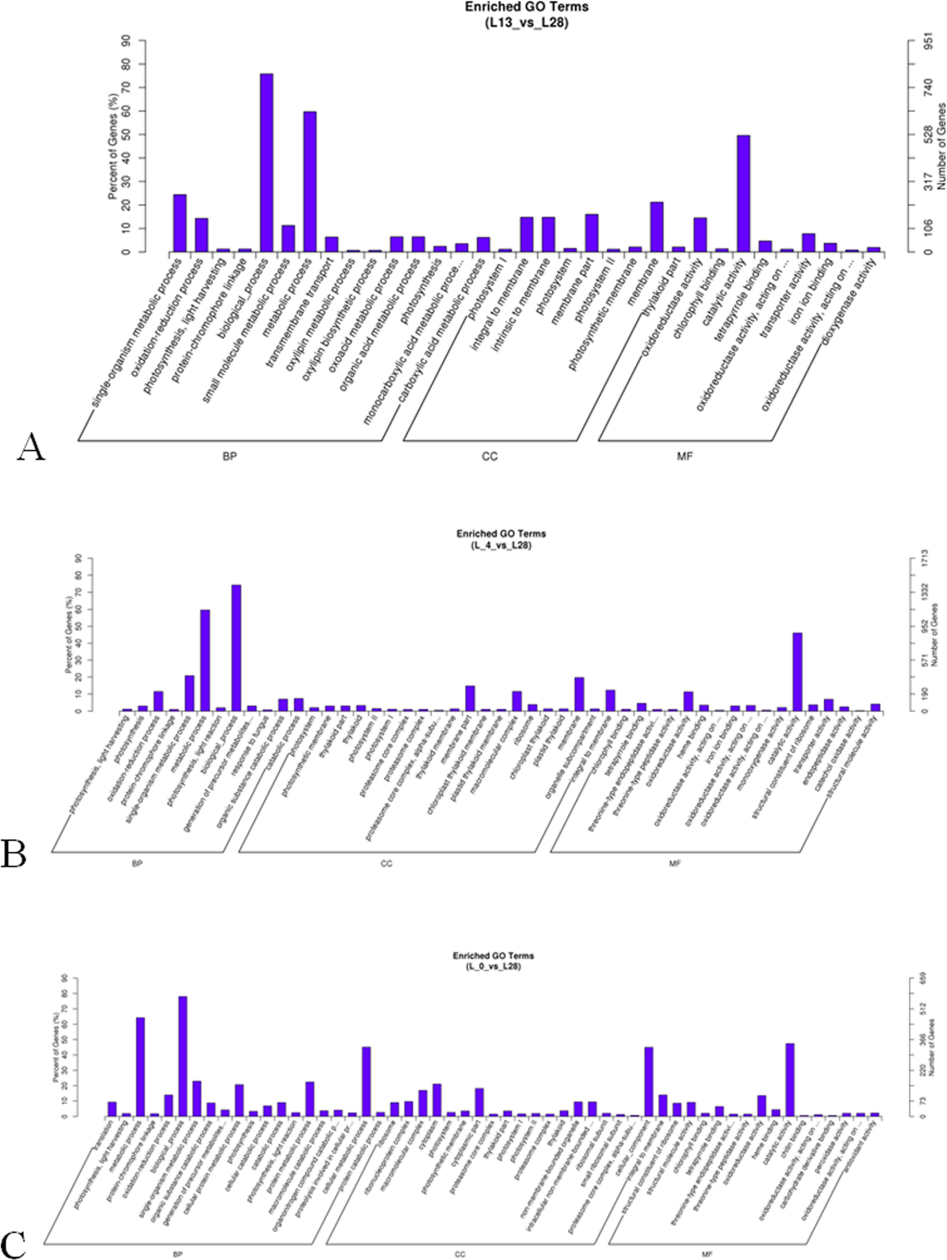
The mRNA GO enrichment analysis showing the differentially expressed genes in three low temperature conditions in the wild banana. Note that the CC group is related to the membrane term. A: L13 *vs* L28; B: L_4 *vs* L28; C: L_0 *vs* L28.

The GO enrichment analysis indicated that all DEGs were assigned to three main GO categories [i.e., biological process (BP), cellular component (CC) and molecular function (MF)] (Figs 1 and S4 and S6 Table). The terms number of three categories was showed in S7 Table.

In the cellular component (CC), 9, 20 and 20 GO terms were significantly enriched in comparison of L13 *vs* L28, L_4 *vs* L28 and L_0 *vs* L28, respectively. Among them, 5, 7 and 2 GO terms were membrane related and significantly enriched in comparison of L13 *vs* L28, L_4 *vs* L28 and L_0 *vs* L28, respectively (Fig 1). Thus, most of the terms were membrane related genes that induced, e.g. the terms of ‘integral to membrane’, ‘intrinsic to membrane’, ‘membrane part’, ‘membrane’ in L13 *vs* L28 (Fig 1A); the terms of ‘membrane part’, ‘membrane’, ‘integral to membrane’ in L_4 *vs* L28 (Fig 1B); the terms of ‘integral to membrane’ in L_0 *vs* L28 (Fig 1C). Further GO enrichment analysis of the wild banana showed the term of ‘photosynthetic membrane’ was all significant differential expression in the three low temperature treatment compared with the control (L_0 *vs* L28, L_4 *vs* L28 and L13 *vs* L28), which indicated photosynthetic membrane was responsive to three low temperature stresses in the wild banana. The GO term of ‘thylakoid membrane’ was significant differential expression only in L_4 *vs* L28, indicating the thylakoid membrane was responded positively to the low temperature (4°C) stress in the wild banana. Thus, based on the above analysis, in the genes enriched in the cellular component (CC) GO terms, the membrane related genes were significant responsive to low temperature stresses in the wild banana.

All of the DEGs were assigned to KEGG pathways (S8 Table), and the top 20 enriched pathways according to the rich factor and the KOBAS software are listed in (S9 Table), with the corresponding details provided in S5 Fig and S10 Table. Among the top 20 enriched pathways, the starch and sucrose metabolism pathway (enriched in L_4 *vs* L_0 and L13 *vs* L28) and the pathways of fatty acid degradation (enriched in L_4 *vs* L28, L_0 *vs* L28 and L13 *vs* L28), biosynthesis of unsaturated fatty acids (enriched in L_4 *vs* L28), fatty acid elongation (enriched in L_4 *vs* L_0) were highly enriched in the wild banana in the low temperature stresses. Yang *et al* (2012) completed an iTRAQ-based comparative proteomic analysis of the temporal responses of plantain to cold stress. They observed that the majority of DE abundant proteins were related to various activities, including carbohydrate metabolic processes, fatty acid beta-oxidation and others, suggesting that an increase in the antioxidant capacity affected the molecular mechanisms underlying the cold tolerance in plantain [46]. In the present research, the pathways of biosynthesis of secondary metabolites (enriched in L_4 *vs* L_0, L_4 *vs* L13, L_0 *vs* L13 and L13 *vs* L28), circadian rhythm-plant (enriched in L_4 *vs* L13, L_0 *vs* L13 and L13 *vs* L28), flavone and flavonol biosynthesis (enriched in L_4 *vs* L28 and L_0 *vs* L28), carotenoid biosynthesis and the sulfur metabolism (enriched in L_0 *vs* L13 and L13 *vs* L28), monoterpenoid biosynthesis (enriched in L_4 *vs* L28 and L13 *vs* L28), arginine and proline metabolism, sesquiterpenoid and triterpenoid biosynthesis (enriched in L_4 *vs* L_0), cutin, suberine and wax biosynthesis (enriched in L_4 *vs* L_0 and L_0 *vs* L28) were highly enriched and responded to the low temperature stresses in the wild banana. These pathways might respond specifically to the three low temperatures in wild banana.

Some pathways showed significant differential expression in the three low temperature stresses when compared with the control (L_0 *vs* L28, L_4 *vs* L28 and L13 *vs* L28), such as the pathways of photosynthesis—antenna proteins, circadian rhythm—plant in L13 *vs* L28; the pathways of photosynthesis—antenna proteins, photosynthesis and proteasome in L_4 *vs* L28; the pathways of ribosome, photosynthesis—antenna proteins, proteasome, glutathione metabolism, tropane and piperidine and pyridine alkaloid biosynthesis in L_0 *vs* L28. These pathways might respond significantly to the three low temperatures in the wild banana. Among them, the photosynthesis—antenna proteins pathway was significant differential expression in all the three low temperature groups. The photosynthesis pathway was significant differential expression in L_4 *vs* L28, suggesting that photosynthesis might respond to the chilling temperature stress in the wild banana. Similarly, photosynthesis was one of the common processes to adapt to cold conditions for banana and plantain [17]. Proteins related the photosynthesis were differential expression responses to cold stress in plantain by a comparative proteomic analysis [46].

#### 3.1.3 Alternative splicing events involved in cold stress of wild banana

**Fig 2:**
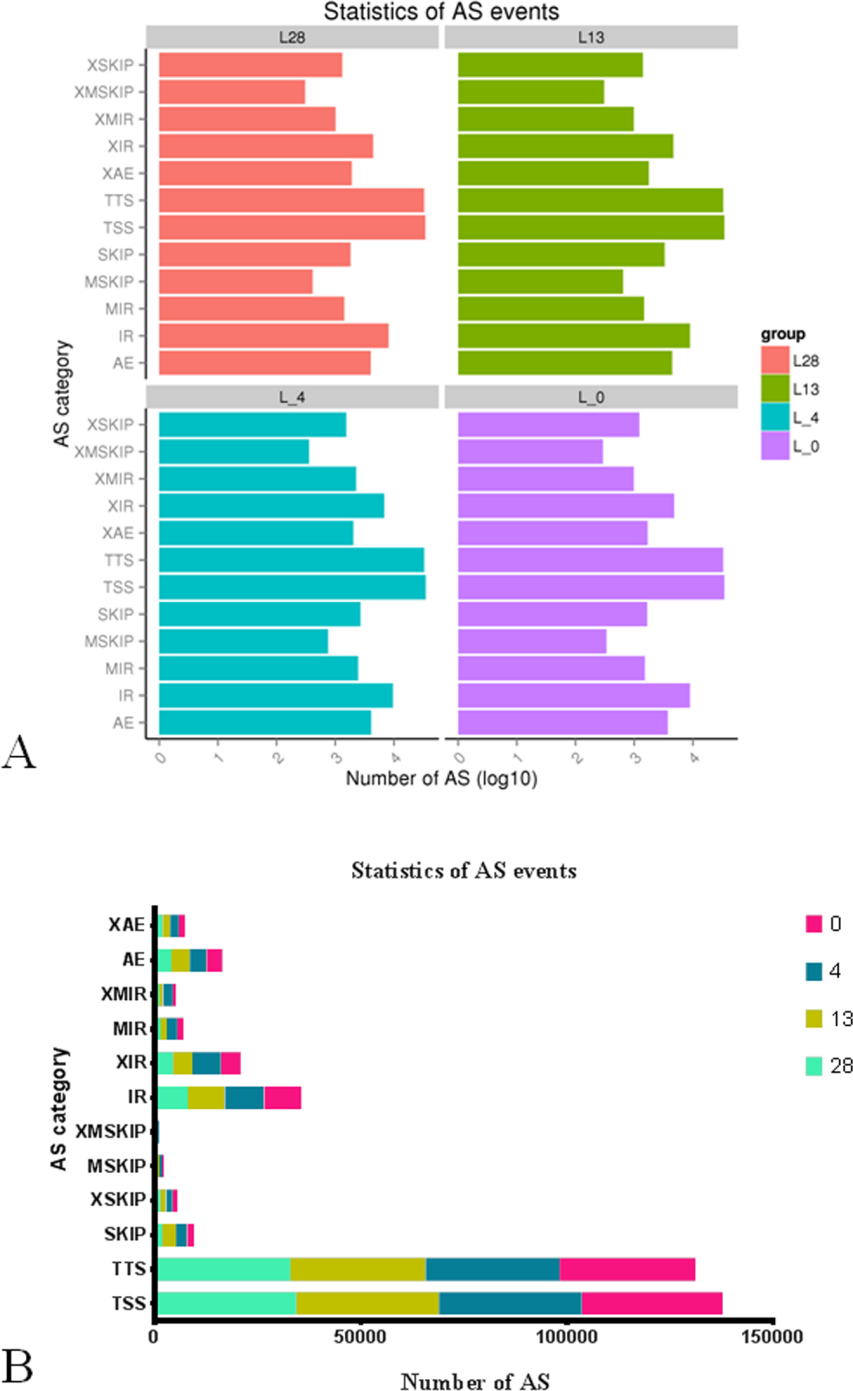
The category and statistics of the AS events showing many AS events accruing in the three low temperature conditions in the wild banana. A: The category of AS events; B: The statistics of AS events.

The alternative splicing (AS) events influenced diverse developmental and physiological processes, including responses to various stresses such as cold and heat stresses. These AS events profoundly affected the re-programming of the transcriptome in response to low temperatures and were divided into three main splicing types: exon skipping, alternative 5’/3’splice sites, and intron retention. Exon skipping included TSS, TTS, SKIP, XSKIP, MSKIP and XMSKIP; intron retention included IR, XIR, MIR and XMIR, and exon skipping, alternative 5’/3’splice sites included AE and XAE. The AS events occurred at different temperatures in the wild banana were shown in Fig 2 and S11 Table.

In the wild banana, the most common AS type was exon skipping (70,844~93,095), followed by intron retention (16,064~21,128), and alternative 5’/3’splice sites (5,477~6,254). Among all the 12 splicing types, TSS and TTS were the most common types (>65%). The distribution of the AS event numbers in the different temperatures were as following: 4°C> 13°C> 0°C> 28°C. There were more AS events at low temperatures (13, 4, 0°C) than at 28°C (control), implying that AS events might be closely related to plant responses to cold conditions. The AS events were found more common in various stress conditions, particularly in the low temperature conditions [47–48] rather than in the normal growing conditions [49–52]. Additionally, more intron retention events were found in the low temperature conditions than that at the normal growing temperature, with the highest number at 4°C. Thus, the intron retention splicing type may play a vital role in wild banana responsive to the low temperature stresses.

### 3.2 Identification of cold responsive lncRNAs in cold stress

#### 3.2.1 Assembly of lncRNA trancripts and identification of lncRNAs

The clean reads were assembled by Cufflinks, and 270,133 assembled transcripts were obtained. The assembly flowchart by cufflinks was shown as S6 Fig.

To identify potential cold responsive lncRNAs in the wild banana, five sequential stringent filters (basic filters) were applied to the 270,133 assembled transcripts. First, the transcripts from four samples were merged using the Cuffcompare program. The 138,992 transcripts detected in at least one sample were selected. Second, transcripts shorter than 200 bp were excluded, which left 97,674 transcripts. Third, the transcripts were filtered base on expression levels (calculated by cufflinks), with the criteria set as FPKM ≥2 for single-exon transcripts and FPKM ≥0.5 for multiple-exon transcripts, after which 92,086 transcripts remained. Fourth, Cuffcompare was used to search the lncRNA databases with the transcripts as queries to identify the known lncRNA. The transcripts that were the same or similar to the known lncRNA and non-mRNA sequences (rRNA, tRNA, snRNA, snoRNA, pre-miRNA, and pseudogenes), were excluded. Transcripts were directly compared with the known lncRNAs and non-coding mRNAs of the species if there were no known lncRNAs in the available databases. A total of 86,779 transcripts remained after the fourth filtering step. Fifth, the known protein-coding mRNAs were excluded, and the candidate lincRNAs, intronic lncRNAs, and anti-sense lncRNAs were classified based on the class code with Cuffcompare (S5A and S5B Fig). The remained 14,034 transcripts after the five sequential stringent filters were considered as candidate lncRNAs, and were subsequently filtered using CPC and PfamScan. The results indicated that 12,462 transcripts satisfied the CPC and Pfam criteria (S5C Fig).

All differential expression transcripts (DETs) (qvalue < 0.05, indicating a difference in expression) including lncRNAs, mRNAs and novel isoforms, were listed in S4 Table, and the annotated lncRNAs were presented in S12 Table.

#### 3.2.2 Prediction of lncRNAs targets

The lncRNAs are a class of regulatory non-coding RNAs that are usually longer than 200 nucleotides. The lncRNAs help regulate a broad range of biological processes in plants, especially reproductive development and responses to stresses. They function at the transcriptional, post-transcriptional or epigenetic levels, either by base pairing (for RNA/DNA sequences) or serving as scaffolds (for proteins). In this study, the *cis*-acting lncRNA targets were predicted (S13 Table). Additionally, the targets 10 kb/100 kb upstream and downstream of the lncRNAs were classified and analyzed as following.

LncRNA targets belonged to transcription factors genes. Many of lncRNA targets were transcription factor (TF) genes, some of which occurred with high frequencies (S14 Table). e.g. the TF genes *zinc finger*, *MYB*, *WD40-repeat-containing domain*, *Helix-loop-helix domain* (*HLH*), *Helix-turn-helix domain* (*HTH*), *C2H2*, *no apical meristem* (*NAM*), *WRKY*, *basic leucine zipper transcription factor* (*bZIP*), *B3 domain-containing transcription factor* (*B3*) and *TCP domain-containing transcription factor* (*TCP*) TFs (frequencies >100), genes of *GRAS*, *squamosa promoter binding protein-box* (*SBP-box*), *DNA-binding-with-one-finger transcription factor* (*dof*), *MADS box*, *auxin response factor* (*ARF)* and *GAGA transcription factor* (*GAGA*) TFs, (frequencies >50), and the genes of *heat shock factor* (*HSF*), *growth regulating factor* (*WRC*), *CCAAT-binding factor*/*nuclear factor-Y* (*CBF*/*NF-Y*), *basic helix-loop-helix domain* (*bHLH*), *NAM*, *ARF*, *transcription factor II B* (*TFIIB*), *root hair defective*, *YABB*, *C3H*, *TATA box binding protein* (*TBP*)-*associated factor II* (*TAFII*), *motif transcription factor* (*DELLA*) and *E2F* (frequencies >10). These results suggested that in the wild banana TF genes targeted by lncRNAs were important for the regulation of the cold-responsive gene expressions.

The *zinc finger* TF reportedly mediate proanthocyanidin biosynthesis [53], while *bHLH* TF appear to regulate anthocyanidin biosynthesis. The *MYB* and *WD repeat-containing protein 40* (*WD40*) TFs can function individually or cooperatively with other TFs (i.e., *MYB-bHLH-WD40 complex*) to control multiple enzymatic steps related to flavonoid biosynthesis in various species [54–55]. The genes encoding these four TFs were the primary TF targets of the lncRNAs, suggesting that lncRNAs were able to affect the wild banana responses to cold stress through the regulation of flavonoid biosynthesis. These finding was also consistent with the results of the KEGG pathway enrichment analysis of the transcriptome and lncRNAs. Moreover, other TF genes that occurred with high frequencies, are also reported to be involved in plant response to cold stress, and some TFs (e.g., *HSFs*) were involved in the wild banana response to cold conditions.

LncRNA targets belonged to non-TF target genes in wild banana. The non-TF genes targeted by the lncRNAs that occurred the highest frequencies encoded *hydrolases*, *protein kinases*, and *domain of unknown function* (*DUF*) proteins (frequencies >1,000) (S14 Table). *ATPases*, *ribosomal proteins*, *tetratricopeptide-like proteins*, *LRRs* (*Leucine-Rich Repeats*), *peptidases* and *armadillo-like helical proteins* (frequencies >500), as well as *sugar-related enzymes*, *cytochromes*, *methyltransferases*, *nucleotide-diphospho-sugar transferases*, *SAM-dependent methyltransferase*, *thioredoxins*, *heat shock proteins*, *histone related*, *major facilitator superfamily domains*, *small GTPase superfamily*, *calcium-binding sites*, *F-box*, *ABC transporters*, *G-proteins*, *calcium-dependent membrane targeting*, *ankyrin repeat-containing domains*, *auxin related*, *ribonuclease H-like domains*, *pectin lyases*, *elongation factors*, *RmlC-like jelly roll folds and peroxidases* (*POXs*) (frequencies >100). Thus, the biochemical processes related to these enzymes, especially the *hydrolases*, *protein kinases*, and *DUF proteins*, were most active in the wild banana during responses to cold stresses. The *DUF proteins*, which comprise domains with uncharacterized functions, were recently reported to influence responses to abiotic stresses in many plants species [56]. On the other side, the biochemical processes associated with the proteins encoded by the other non-TF gene targets of the lncRNAs affected important cellular activities. For example, ATPases contributed to energy release; ribosomal proteins mediated protein biosynthesis and DNA repair; tetratricopeptide-like helical proteins helped regulate the cell cycle, transcription and protein folding; LRRs functioned as protein kinases and are involved in responses to biotic and abiotic stresses; peptidases hydrolyzeed peptides into amino acids; and armadillo-like helical proteins [e.g., casein kinase II (CKII)] mediated phosphorylation. Additionally, the biochemical processes associated with the sugar-related enzymes, cytochromes, methyltransferases, nucleotide-diphospho-sugar transferases, SAM-dependent methyltransferase, thioredoxins, HSPs, histone related, major facilitator superfamily domains, small GTPase superfamily, calcium-binding sites, F-box, ABC transporters, G-proteins, calcium-dependent membrane targeting, ankyrin repeat-containing domains, auxin related, ribonuclease H-like domains, pectin lyases, elongation factors, rmlc-like jelly roll folds and POXs (i.e., sugar metabolism and transport, epigenetic modifications, calcium signaling and redox reaction) were also regulated by the lncRNAs in the wild banana during responses to cold stress.

The fact that many *protein kinase* genes were targeted by the lncRNAs in the wild banana responses to cold stress was very interesting. *Protein kinases* were divided into the following three broad classes: *serine*/*threonine-protein kinases*, *tyrosine*-*protein kinases* and *dual specificity protein kinases*. *Serine*/*threonine protein kinases* phosphorylated serine or threonine residues in their substrates, resulting in changes of enzymatic activity, which was an example of the very important posttranslational modification. *Tyrosine*-*protein kinases* could transfer a phosphate group from ATP to a tyrosine residue in a protein, usually leading to a functional change due to modified enzymatic activity, cellular location, or association with other proteins. These changes could have implications for signal transduction pathways as well as the activation of TFs. *Dual specificity protein kinases*, including MEKs (MAPK, MAPKK) phosphorylated both threonine and tyrosine in target proteins. Among the non-TF genes targeted by the lncRNAs, 1,737 encoded protein kinases, with 1,364 *serine*/*threonine protein kinases*, 541 *tyrosine-protein kinases* and 1,208 *dual specificity protein kinases* (S14 Table). This distribution of protein kinases suggested the lncRNAs affected the wild banana responses to cold conditions via mainly the regulation of *serine*/*threonine* or *dual specificity protein kinases*. Therefore, during the wild banana responses to cold stress, the lncRNAs would target many protein kinases to alter enzyme activity, cellular location, or associations with other proteins during post-translational modifications, signal transduction pathways and TFs activation.

Finally, some previously reported cold-related genes were found to be targeted by the lncRNAs in the wild banana response to cold stress, including the genes of *WCOR413* (cold responsive gene 13 in wheat), stress up-regulated *Nod 19* (*nodulation 19*), and a *stress-responsive protein*. Moreover, some genes related to HAC/HDAC (Histone acetylation/Histone Deacetylation), the circadian rhythm and AS events, such as *sin3*, *ada1*/*Tada1*, *CKII*, *Lsm*, were also targeted by the lncRNAs during wild banana responses to cold stress.

#### 3.2.3 The targets GO and KEGG enrichment analysis of differential expression lncRNAs involved in cold stress

The results of the GO enrichment analysis of the differential expression lncRNAs (DELs) revealed a lack of any significant enrichment (P< 0.05 for statistical significance) (S15 Table). However, many KEGG pathways were enriched among the DELs targets (S16 Table), and the top 20 enriched pathways according to the rich factor were listed in S17 and S18 Tables, S7 Fig.

Among the top 20 enriched pathways, one was detected in all six groups (L_0 *vs* L28, L13 *vs* L28, L_0 *vs* L13, L_4 *vs* L13, L_4 *vs* L28 and L_4 *vs* L_0). Additionally, the glycolysis/gluconeogenesis pathway was enriched in five of six groups, with the exception being L_0 *vs* L13. The spliceosome, carbon metabolism and biotin metabolism pathways were enriched in four groups. The citrate cycle (TCA cycle), arginine and proline metabolism, sulfur relay system, galactose metabolism, flavonoid biosynthesis, diterpenoid biosynthesis, aminoacyl-tRNA biosynthesis, proteasome, and ribosome biogenesis in eukaryotes pathways were enriched in three groups. Specifically, the TCA cycle and sulfur relay system pathways were enriched in the three groups of L_4 *vs* L13, L_4 *vs* L28 and L_4 *vs* L_0, which were all related to the chilling temperature (4°C). Thus, the chilling temperature was expected to induce the lncRNAs to regulate the TCA cycle and sulfur relay system pathways. Moreover, the flavonoid biosynthesis and diterpenoid biosynthesis pathways were enriched in the three groups of L_4 *vs* L13, L13 *vs* L28 and L_0 *vs* L13, which were all related to the critical growth temperature (13°C), suggesting that the critical growth temperature stimulated the lncRNA to regulate the flavonoid biosynthesis pathways.

The diterpenoid biosynthesis pathways, the thiamine metabolism pathway, folate biosynthesis, fatty acid biosynthesis, oxidative phosphorylation, propanoate metabolism, fructose and mannose metabolism, biosynthesis of secondary metabolites, selenocompound metabolism, zeatin biosynthesis, glyoxylate and dicarboxylate metabolism, beta-Alanine metabolism, RNA transport, C5-Branched dibasic acid metabolism, glycosyl-phosphatidylinositol (GPI)-anchor biosynthesis, protein export, alpha-linolenic acid metabolism, ABC transporters, biosynthesis of amino acids, base excision repair, stilbenoid, diarylheptanoid and gingerol biosynthesis pathway, were enriched in two groups; 36 pathways were enriched in only one of the six groups. The tropane, piperidine and pyridine alkaloid biosynthesis, isoquinoline alkaloid biosynthesis, glycine, serine and threonine metabolism, tyrosine metabolism, lysine biosynthesis and photosynthesis-antenna proteins pathways were enriched only in the L_4 *vs* L_0 group. The sesquiterpenoid and triterpenoid biosynthesis, nucleotide excision repair, and other types of O-glycan biosynthesis pathway were enriched only in the L_4 *vs* L13 group. The pathways of the pentose phosphate pathway (PPP), RNA polymerase, peroxisome, and alanine, aspartate and glutamate metabolism were enriched only in the L_4 *vs* L28 group. The pathways of the circadian rhythm-plant, linoleic acid metabolism, SNARE (Soluble NSF attachment protein receptor) interactions in vesicular transport, histidine metabolism, phenylalanine metabolism, biosynthesis of unsaturated fatty acids, glycerolipid metabolism, phenylpropanoid biosynthesis, pantothenate and CoA biosynthesis, phagosome, protein processing in endoplasmic reticulum were enriched only in the L_0 *vs* L13 group. The pathways of the purine metabolism, pyruvate metabolism, phosphatidylinositol signaling system, non-homologous end-joining, 2-oxocarboxylic acid metabolism, ubiquitin mediated proteolysis, were enriched only in the L_0 *vs* L28 group. The pathways of the sulfur metabolism, RNA degradation, sphingolipid metabolism, amino sugar and nucleotide sugar metabolism, synthesis and degradation of ketone bodies and endocytosis were enriched only in the L13 *vs* L28 group. The above analyses indicated those pathways might respond to cold stresses and be regulated by the lncRNAs in the wild banana.

### 3.3 Comparative analysis of the expression levels and structures between the total lncRNAs and mRNAs

#### 3.3.1 Comparative analysis between the total lncRNAs and mRNAs expression levels

**Fig 3:**
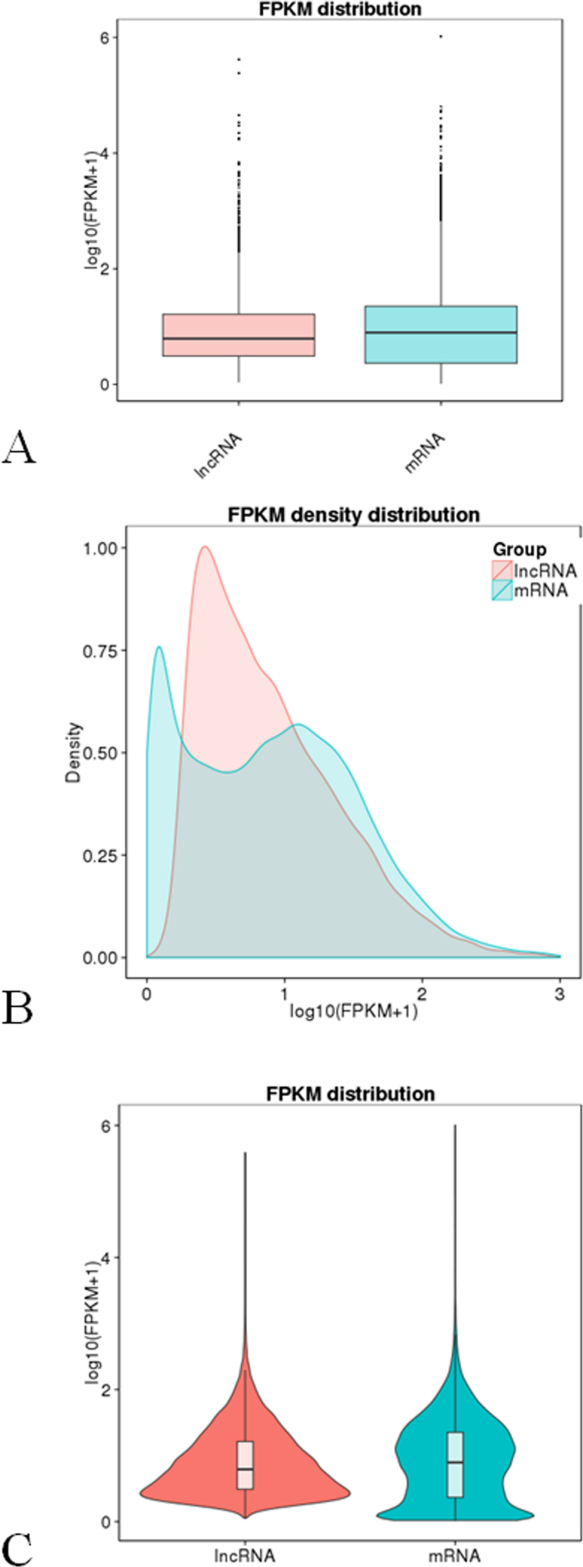
The comparisons of the FPKM values between lncRNAs and mRNAs showing a relative higher mRNA expression level and a lower lncRNA level. A: FPKM distribution; B: Density; C: Expression of the violin.

Cuffdiff was used to analyze the lncRNAs and mRNAs expression levels. The lncRNA and mRNA mean sequence reads for all treatment groups were normalized to FPKM values, and the log_10_ (FPKM+1) values were used to draw box plots (Panel A, B in Fig 3) and the violin (Panel C in Fig 3). Overall, the lncRNA expression levels were lower than the protein-coding mRNA expression levels.

#### 3.3.2 The structure comparisons of lncRNAs and mRNAs

**Fig 4:**
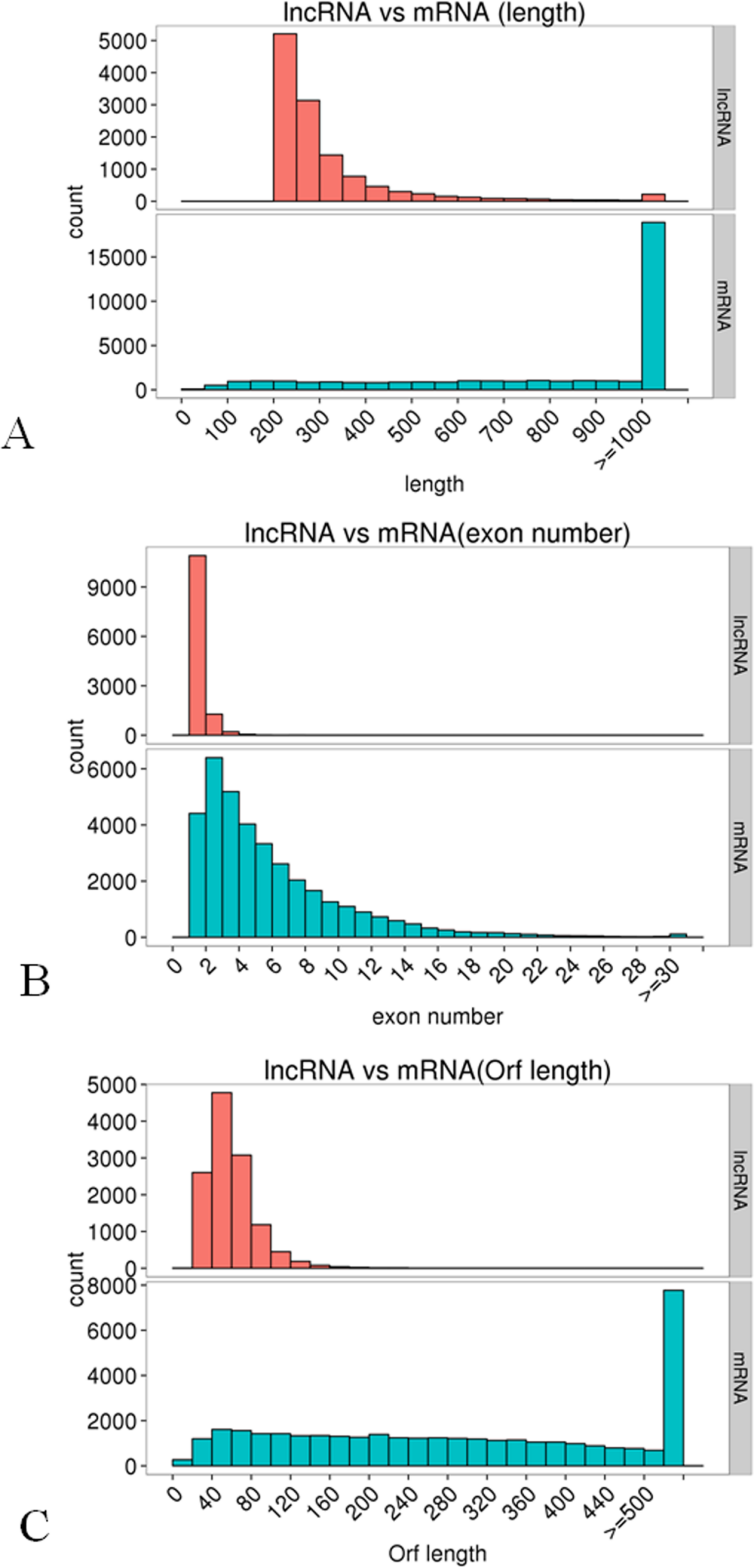
**The structure comparisons between lncRNAs and mRNAs in the wild banana showing the length of lncRNA was shorter than that of mRNA (A), exon number of lncRNA were less than that of mRNA (B), and ORF length of lncRNA was shorter than that of mRNA.** A: mRNA *vs* lncRNA in length; B: mRNA *vs* lncRNA in exon number; C: mRNA *vs* lncRNA in ORF length.

Transcript lengths, number of exons, and ORF lengths were provided in Fig 4. Most of the mRNAs were longer than 1000 bp, while the lncRNAs were 200–500 bp long. Additionally, most of the lncRNAs consisted of 1–4 exons, unlike most of the mRNAs, which comprised 1–32 exons. The ORF lengths were 40–120 bp for most of the lncRNAs, but exceeded 500 bp for most of the mRNAs. Therefore, the structural characteristics of the predicted wild banana lncRNAs were consistent with those of generally known lncRNAs.

### 3.4 Comparative analysis of the expression levels and chromosome distributions of the differential expression transcripts (DETs)

#### 3.4.1 The comparative analysis of the expression levels of DETs

**Fig 5:**
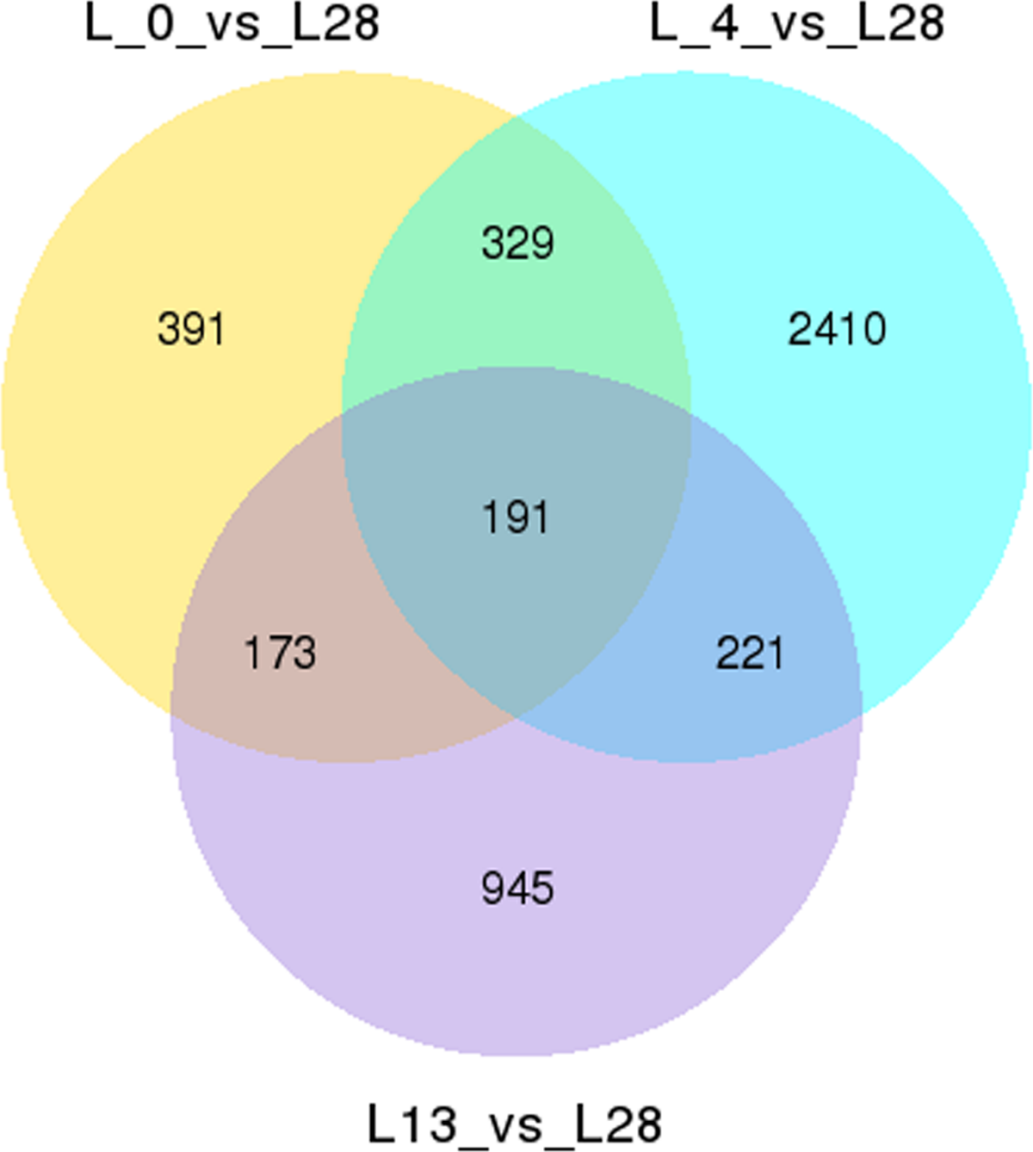
The venn diagrams analysis of the number of DETs in the three low temperature treatment groups in the wild banana showing significantly more DET numbers in the 4°C group.

Volcano plots revealed the DETs in all the six groups (L_0 *vs* L28, L13 *vs* L28, L_0 *vs* L13, L_4 *vs* L13, L_4 *vs* L28 and L_4 *vs* L_0) (S8 Fig). The L_4 *vs* L13 group consisted of the most up- and down-regulated transcripts with 1,572 and 1,845 transcripts, respectively. The L_4 *vs* L28 group contained the second most up- and down-regulated transcripts. These observations implied that 4°C might be the key temperature for DETs during the wild banana responses to cold stress. Venn diagrams for the DETs (Figs 5 and S9) indicated that the L_4 *vs* L28 group was associated with the most DETs, implying wild banana was significantly responsive to cold stress at 4°C.

Based on the cluster analysis of the DETs (S10 Fig and S1 File), all DETs were assigned to five main clades, which exhibited five patterns of DETs changes in the cold-stressed wild banana. The DETs at 4°C were assigned to a single clade, which included obviously up- or down-regulated DETs. The corresponding DETs in the other temperature groups exhibited the opposite patterns, suggesting the important and specific effects induced by the 4°C treatment.

#### 3.4.2 The chromosome distribution of DETs

**Fig 6:**
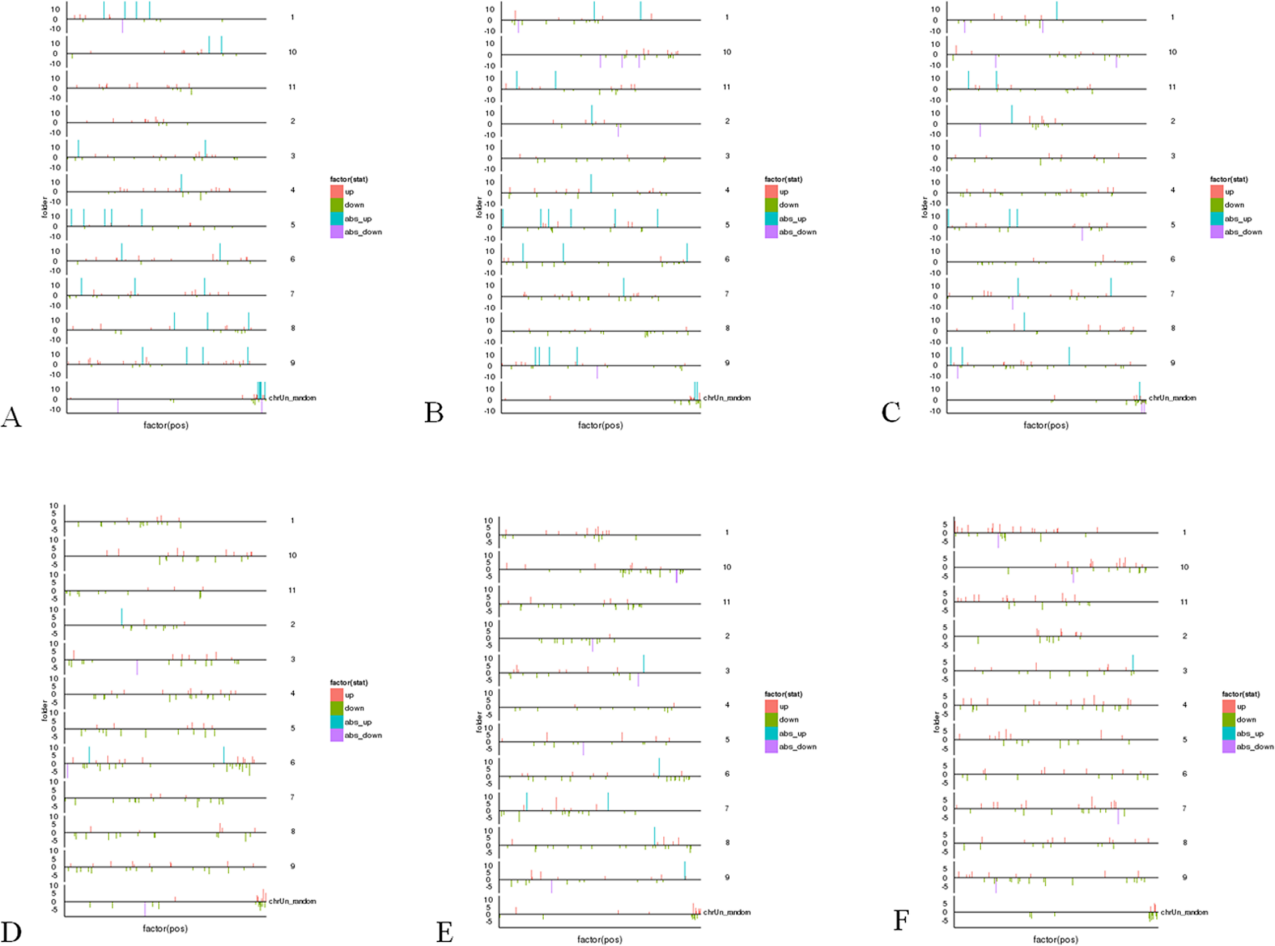
The DETs distribution in the chromosomes of the wild banana showing during cold stress, especially at 4°C, there were more absolute up- and down-regulation DETs. A: L_4 vs L_0; B: L_4 vs L13; C: L_4 vs L28; D: L_0 vs L13; E: L_0 vs L28; F: L13 vs L28.

The chromosomal distributions of the DETs were presented in Fig 6, and the up- or down-regulated transcripts were summarized in S19 Table. The L_4 *vs* L28 group contained the most absolutely up- regulated (304) or down-regulated (79) transcripts, followed by L_4 *vs* L13 and L_4 *vs* L_0 groups. The lowest number of absolutely up- regulated (11) and down-regulated (16) transcripts was in the L13 *vs* L28 group. Interestingly, the number of absolutely up- and down-regulated transcripts in 4°C related groups (L_4 *vs* L13, L_4 *vs* L28 and L_4 *vs* L_0) were significant more than the other temperature treatment groups (L_0 *vs* L28, L13 *vs* L28 and L_0 *vs* L13). Thus, the chilling temperature (4°C) might be the key temperature for DETs during the wild banana responses to cold stress.

### 3.5 Validation of DE mRNA and relationships between lncRNAs and the targets by qPCR during cold stress

#### 3.5.1 Validation of DE mRNA of protein-coding genes by qPCR

**Fig 7:**
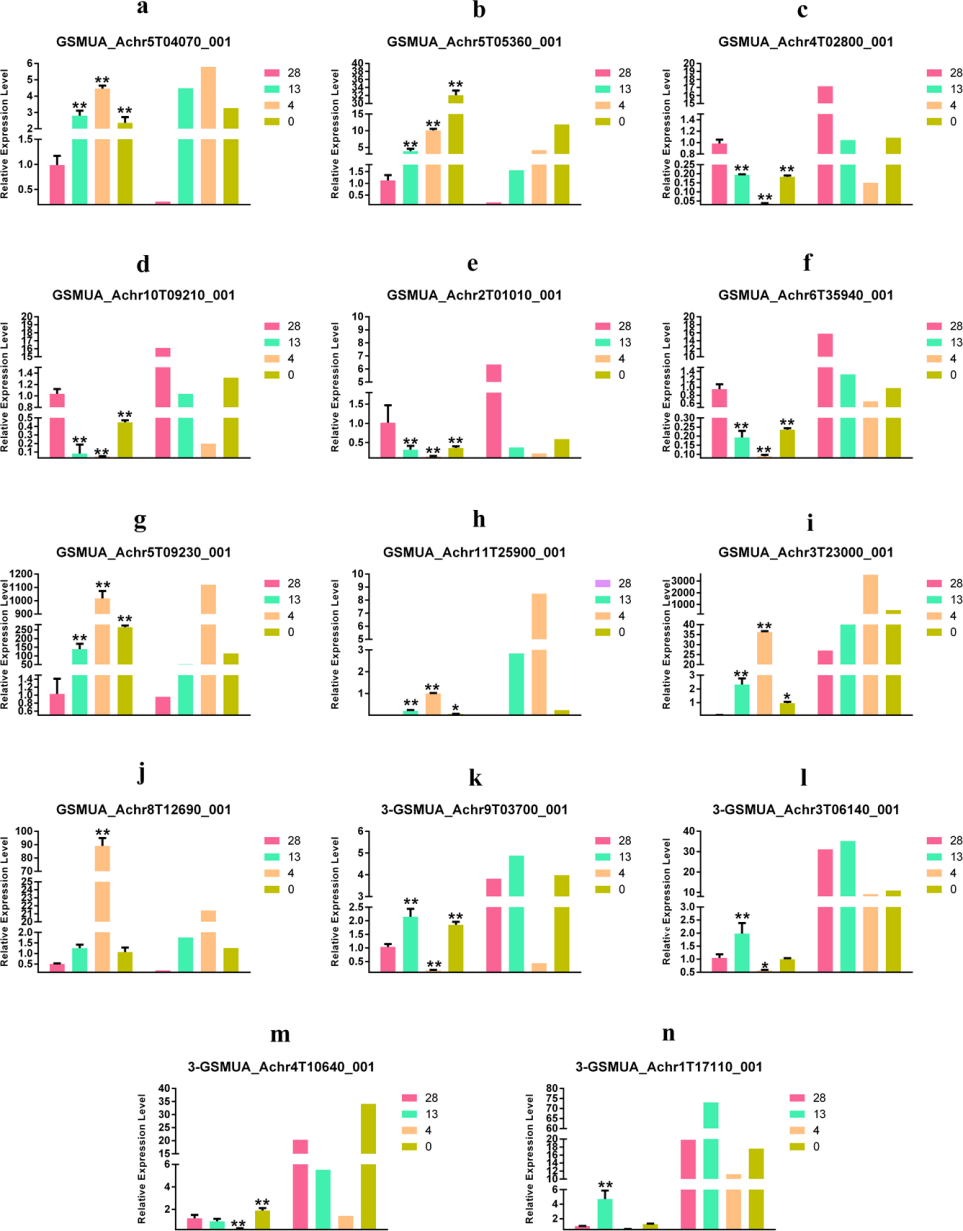
Validation of DEGs by qPCR (comparing with FPKM values) during cold stress in the wild banana showing the FPKM data confirmed by qPCR tests.

To validate the mRNA profiles obtained by RNA-seq, a qPCR analysis was completed with 20 selected DEGs with comparisons with FPKM values (Fig 7). The FPKM values for the mRNAs, lncRNAs and novel isoforms were calculated with Cuffdiff (S3 Table). Although there were some quantitative differences in expression levels, the expression patterns based on the qPCR and RNA-seq data were identical for 14 of the 20 genes tested, which included the genes of *K*^*+*^ *potassium transporter*, *multicopper oxidase*, *NAD-dependent epimerase/dehydratase*, *calcium-dependent lipid-binding transcriptional regulator*, *DUF4057*, *PWRKY TF-group IIc*, *peptidase S8*, *pyridoxal phosphate-dependent transferase*, *CKII*, *calcium-binding EF-hand*, *ammonium transporter*, and 3 genes without matches in the database, i.e., GSMUA_Achr1T17110_001, GSMUA_Achr2T01010_001 and GSMUA_Achr 5G09230_001. The qPCR and RNA-seq expression patterns of the other six genes were also observed, and differences were only found at 0°C related groups, which included the genes of *ChaC-like protein*, *fatty acid hydroxylase*, *short-chain dehydrogenase/reductase SDR*, and 3 genes without matches in the database, i.e., GSMUA_Achr3G23440_001, GSMUA_Achr9G15270_001 and GSMUA_Achr11G25900_001. Thus, the RNA-seq data were considered to be reliable.

#### 3.5.2 Validation of cold stress-responsive lncRNA and targets by qPCR in the wild banana

**Fig 8:**
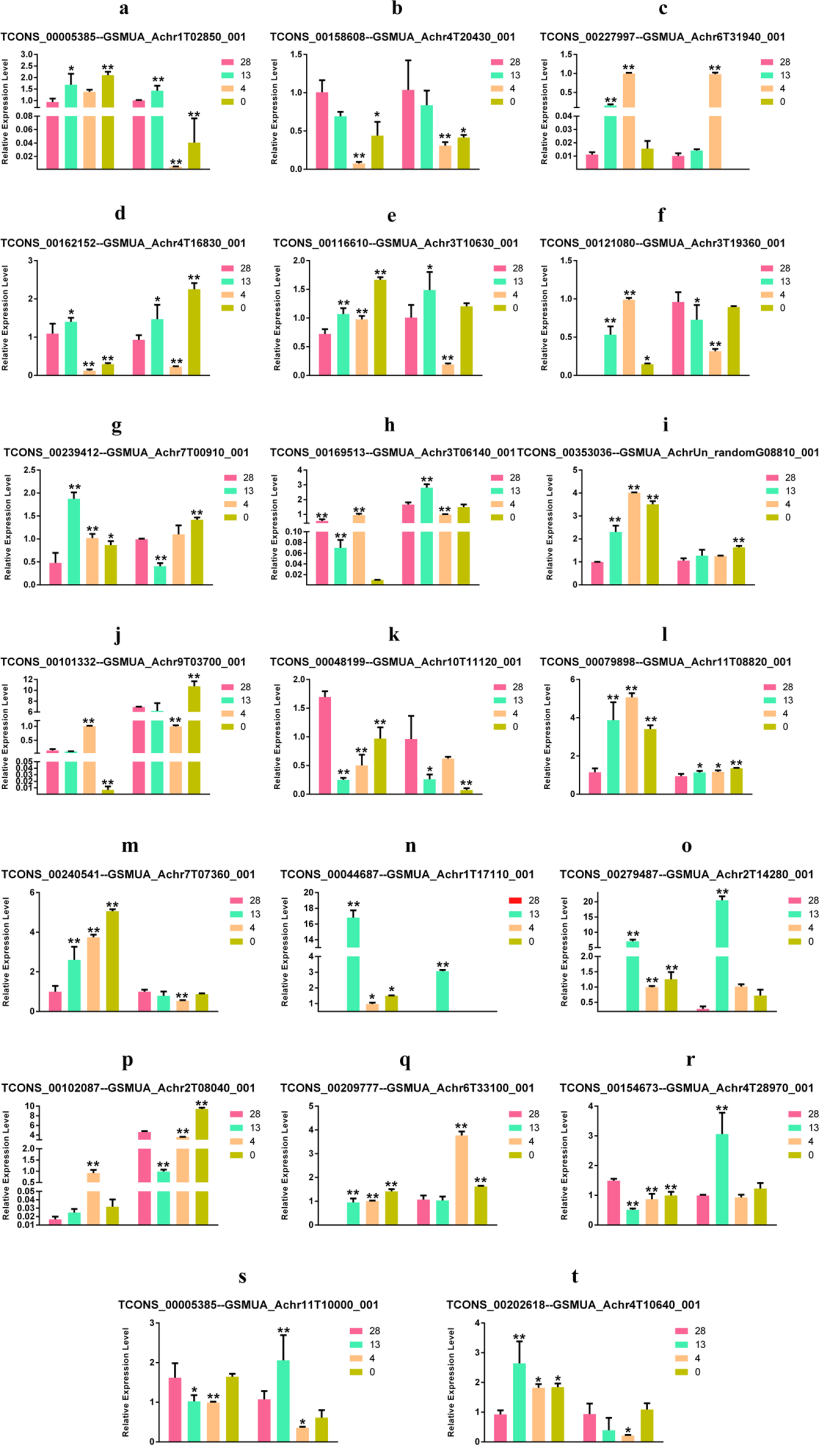
Validation of DE lncRNAs and their target genes by means of qPCR in the cold stress conditions in the wild banana showing positive, negative and positive or negative mixed regulations of the lncRNAs.

To validate the relationships between the cold responsive lncRNAs and the targets, 20 cold responsive lncRNAs and the corresponding targets were analyzed by qPCR (Fig 8). The results revealed the complexity of the regulatory relationships between the lncRNAs and the corresponding targets. A positive regulation in all the cold treatments was observed for 5 of the 20 lncRNA and target pairs (Panel a, b, c, d, e in Fig 8), while a negative regulation in all the cold treatments was detected for three of the 20 lncRNA and target pairs (Panel f, g, h in Fig 8). Meanwhile, a positive or negative mixed regulation was observed only in response to specific cold treatments for the remaining 12 lncRNA and target pairs (Panel i, j, k, l, m, n, o, p, q, r, s, t in Fig 8). These results implied that some lncRNAs regulated their targets in all cold stress conditions, whereas some other lncRNAs regulated their targets only in specific temperature conditions. However, all of the lncRNAs regulated their targets either positively or negatively.

## 4 Discussion

In the present research, mRNA and lncRNAs were systematically identified and analyzed of the wild banana in different cold stress conditions, i.e. 13°C (critical growth temperature), 4°C (chilling temperature), 0°C (freezing temperature) compared with 28°C (the normal growing condition, control). Up the date, this is the first report on the global identification the lncRNAs responsive to cold stresses in the wild banana. Notably, total 12,462 high-confidence lncRNAs were identified based on stringent criteria. Although several parameters may affect the efficiency of lncRNA identification, such as genotypes, reference genome used and sample treatment conditions, the high throughput deep sequencing by RNA-seq employed in the present research revealed details of the lncRNAs information. In other plants, lncRNAs were also identified, e.g. kiwifruit (14,845) [57], three tetraploid wheat (59,110 in Kiziltan, 57,944 in TR39477 and 40,858 in TTD-22) [58], precocious trifoliate orange (6,584) [59], maize (20,000) [60], *Brassi napus* (3,181) [61], rice (2,224) [62] and *Brachypodium distachyon* (5,851, 2,681 and 15,948 depending on the reference genome used) [63]. It was found that more lncRNAs were observed when more tissues were sampled and the depth of sequencing was increased [58, 63–70].

The results of comparative analysis between the total lncRNAs and mRNAs expression levels showed the lncRNAs were expressed at low levels compared with mRNAs. Similar results were observed for lncRNAs in other plant species, such as cassava [32], cucumber [24], maize [60] and orange [59].

Over 20% of genes were alternatively spliced in *Arabidopsis* and rice, and the most prevalent type is intron retention [70]. The alternative splicing may participate in new molecular pathways [71–72]. A previous study of trifoliate orange indicated *PtFLC* was regulated post-transcriptionally by alternative splicing (exon skipping) and formed five splice variants. The alternative splicing pattern of *PtFLC* was altered through developmental stages and further influenced by temperature fluctuations [73]. There were much more alternatively spliced events occurred in the wild banana in low temperature stresses. And the intron retention was the most prevalent alternatively spliced type in the chilling stress temperature (4°C). It has been reported that alternatively spliced events involved in responses to cold stress in plant [47–48]. These suggest the alternatively spliced events may be influenced by low temperature, and the molecular mechanisms need further investigation.

Based on the GO enrichment analyses of DEGs, we found that many membrane related genes (especially the photosynthetic membrane and thylakoid membrane) were differentially expressed in the cold stress conditions. The growth and development of plants and their internal structures were affected in chilling stress. Chloroplast was thought to be the centrality as its sensitivity to chilling stress [74], and was the earliest affected by chilling injury [75]. When chilling injury was occurred, similar effects that the first manifestations were chloroplast swelling, a distortion and swelling of thylakoids, were reported by Wise et al. [76]. In the chloroplasts as the organelles for photosynthesis, of which the photosynthetic membrane was thought to be the most complex and ingeniously constructed of all biological membranes, would capture light quanta and drive a series of redox reactions when the conditions of light and temperature changed [77]. The primary reactions of photosynthesis occured in thylakoid membranes, which were contained in the chloroplast [78]. During chilling, thylakoid membranes were distorted at the first stage. The chloroplast envelope underwent disintegration at the second stage. The thylakoid intraspaces were dilated at the third stage [76, 79–81]. The thylakoid membranes of one cold-hardy species, *Spinacia oleracea*, were investigated in cold hardening temperatures. The paracrystalline array of proteins in thylakoid membranes were formed, and a decreased particle concentration on the inner fracture face of the thylakoid membranes was observed in cold-acclimated plants, which was a homogenization of particle size in membranes of nonacclimated thylakoids [82]. The peripheral reticulum with the inner membrane of the chloroplast envelope supported metabolite transport [83–84], and the transport was controlled by the membrane [85]. Wise et al. (1983) proposed the peripheral reticulum develops to increase inner chloroplast membrane surface area during a chilling-induced decrease in transport capacity [76]. In the wild banana, the cold stress might influence the photosynthesis, but the membrane related genes were considered to respond significantly to the cold stress to guarantee the photosynthesis. As a result, the wild banana was able to grow well in relatively low temperature conditions.

In response to the abiotic stresses occurred in the plants, plants were able to adapt to, avoid and overcome the problems caused by the stresses by means of various physiological and biochemical mechanisms. Among them, plant cuticular wax layer provided a protective barrier against the water loss, and the cuticle layer plays a key role in maintaining the plant’s integrity as the primary interface [86]. The production of wax was affected by temperature in plants, and more waxes were produced at lower temperatures in plants such as *Brassica* [87]. The quantities of cuticular wax were reduced on the third leaf of maize exposed to cold stress [88]. A series of stresses influenced cuticular waxes, and commonly changed the amount and composition of wax [86]. The above reports of cuticular wax and cuticle as the plant responds to water deficient stress are in agreement with the KEGG enrichment analysis results of the present research in the wild banana. The ‘cutin, suberine and wax biosynthesis’ pathway was enriched in groups of L13 *vs* L28, L_4 *vs* L28 and L_0 *vs* L28, and especially on top 20 pathway in L_0 *vs* L28. Many genes in this pathway were activity in the investigated three low temperature conditions in the wild banana. Interestingly however, the expressions of *cytochrome P450* family members in ‘biosynthesis of unsaturated fatty acids’ and ‘cutin and suberin biosynthesis’ were up-regulated in L_4 *vs* L28, but down-regulated in L13 *vs* L28 and L_0 *vs* L28. In contrast, the *fatty acyl-CoA reductase* family members in ‘wax biosynthesis’ were up-regulated in L13 *vs* L28 and L_0 *vs* L28, but down-regulated in L_4 *vs* L28. In addition, these two genes were the first key gene for these pathways. Membrane properties would be immediately altered, which did not survive in its simplest form, and finally, transport processes across membranes would be interrupted by lowered temperatures as well [89–90]. The water diffusion would be induced by the change of cell membrane permeability. And the water deficiency contribute to the wax synthesis, and cutin biosynthesis [91]. Thus, via a sires of gene regulations, armed with a protective cutin, cuticular waxes, a primary barrier to water loss and membrane-water combination required for water retention of the wild banana was finally established when low temperature stresses were encountered.

In summary, the present study provided a comprehensive resource for the wild banana response to cold stresses from the aspect of transcriptome and lncRNAs. It would be useful to find target genes for cold-resistant breeding of cultivated banana plants. More work was needed to validate the candidate genes, pathways and alternative splicing events, which were significantly differentially expressed in various cold stress conditions.

## 5. Conclusions

In this study, we examined cold-responsive mRNAs and lncRNAs in the cold-stressed wild banana by RNA-seq. We identified 12,462 lncRNAs in cold-stressed wild banana. Additionally, much more alternative splicing events occurred in the wild banana in the cold stress conditions, and demonstrated possible molecular mechanisms. The membrane related genes responded positively to the low temperature stresses. The pathways might respond specifically to the different low temperatures, such as photosynthesis-antenna proteins; photosynthesis; circadian rhythm-plant; glutathione metabolism; starch and sucrose metabolism; cutin, suberine and wax biosynthesis. In 4°C comparison of the control, the wild banana contained the most absolutely up- regulated (304) and down-regulated (79) transcripts. The results of the present research provide necessary information for cold resistance breeding in banana.

## Supporting information

S1 Fig The leaves phenotypes of the wild banana and ‘Tianbaojiao’ at different low temperatures stress. (TIF)

S2 Fig The Bioinformatic pipeline. (TIF)

S3 Fig The FPKM distribution box-plot and density distribution plot of all the transcripts. (TIF)

S4 Fig The mRNA GO enrichment analysis. (TIF)

S5 Fig The scatter diagrams of the top 20 enriched KEGG pathways of the targets for DE mRNAs mapped to Genome A. (TIF)

S6 Fig The changes of transcripts in the process of basic filter and coding-protein filter by CPC and PFAM. (TIF)

S7 Fig The scatter diagrams of the top 20 enriched KEGG pathways of the targets for the DE lncRNAs mapped to Genome A. (TIF)

S8 Fig Volcano plot of the different expressed transcripts. (TIF)

S9 Fig The Venn diagrams of the different expressed transcripts. (TIF)

S10 Fig The cluster analysis of the different expressed transcripts. (TIF)

S1 Table The primers used in this study. (XLS)

S2 Table The results of mRNA and lncRNA from four wild banana libraries mapped to genome A. (XLS)

S3 Table The FPKM of the mRNA, lncRNA and novel isoform mapped to Banana Genome A. (XLS)

S4 Table Detailed information of the different expressed mRNA, lncRNA and novel isoform mapped to Banana Genome A. (XLS)

S5 Table Annotation of different expression mRNA and novel isoform in six groups mapped to Banana Genome A. (XLS)

S6 Table Significant GO terms for mRNAs mapped to Banana Genome A. (XLS)

S7 Table The numbers of terms enriched in DEGs in the six groups during cold stress in the wild banana. (XLS)

S8 Table KEGG enrichment analysis of mRNA mapped to Banana Genome A. (XLS)

S9 Table The top 20 enriched pathways of mRNA mapped to Banana Genome A. (XLS)

S10 Table The top 20 KEGG pathways different expressed mRNA in the six groups during cold stress in the wild banana. (XLS)

S11 Table The AS events numbers in four libraries of wild banana. (XLS)

S12 Table Annotation of different expressed lncRNA in six groups mapped to Banana Genome A. (XLS)

S13 Table Annotation of lncRNA targets mapped to Banana Genome A. (XLS)

S14 Table Target genes for lncRNA during cold stress in the wild banana. (XLS)

S15 Table Significant GO terms for target genes of different expressed lncRNAs mapped to Banana Genome A. (XLS)

S16 Table KEGG enrichment analysis of lncRNA mapped to Banana Genome A. (XLS)

S17 Table The top 20 enriched pathways of lncRNA mapped to Banana Genome A. (XLS)

S18 Table The top 20 KEGG pathways DELs targets in the six groups during cold stress in the wild banana. (XLS)

S19 Table The numbers of absolutely up/down-regulated transcripts distributions on the chromosomes during cold stress in the wild banana. (XLS)

S1 File The cluster analysis of DETs in detail. (PDF)
