# Supplementary figures and images for: Genome-wide identification and characterization of mRNAs and lncRNAs involved in cold stress in the wild banana (Musa itinerans)
Source: PLoS One. 2018 Jul 9;13(7):e0200002. doi: 10.1371/journal.pone.0200002 (PMC6037364; doi:10.1371/journal.pone.0200002)

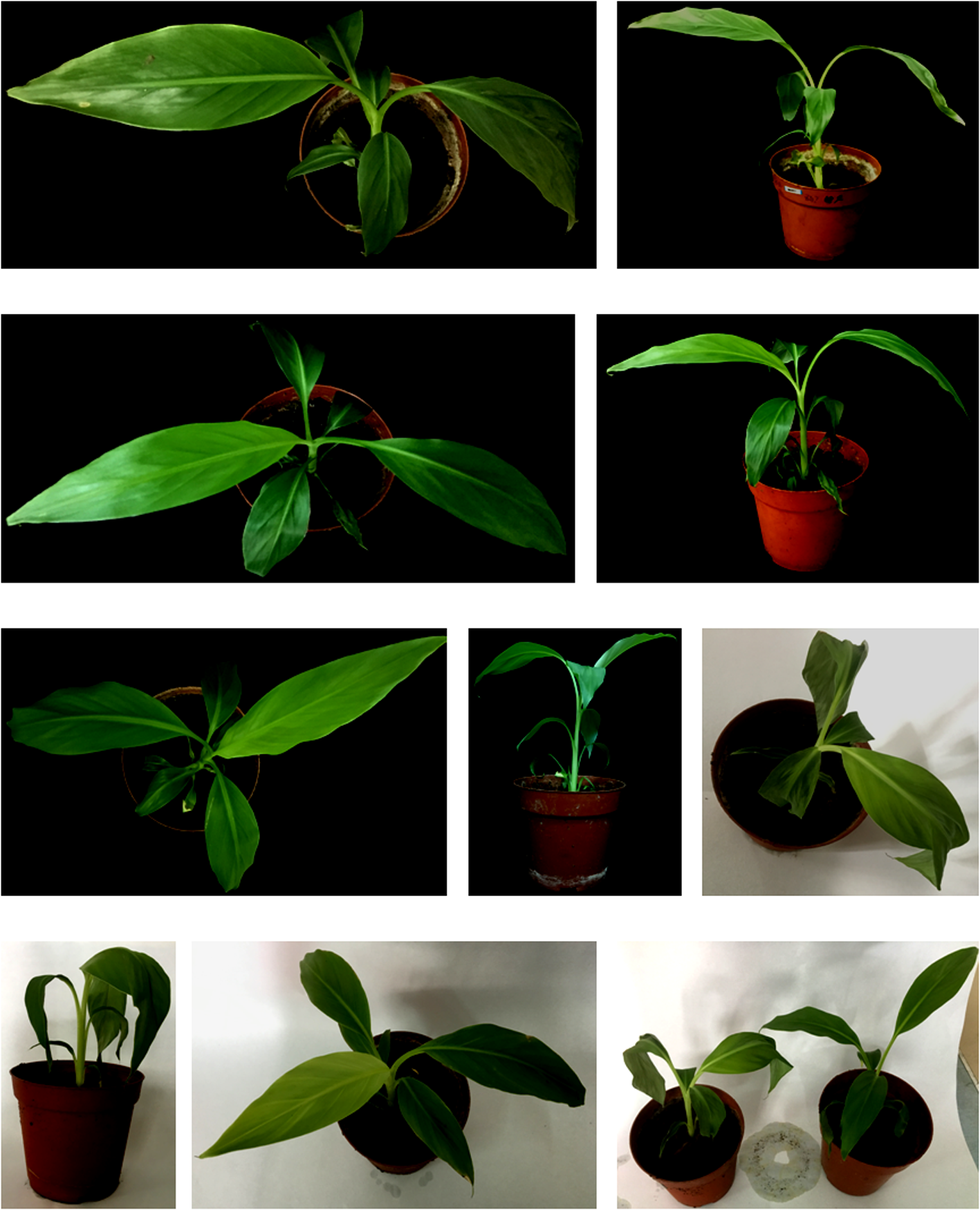

Supplement: S1 Fig — (TIF) [file pone.0200002.s001.tif]

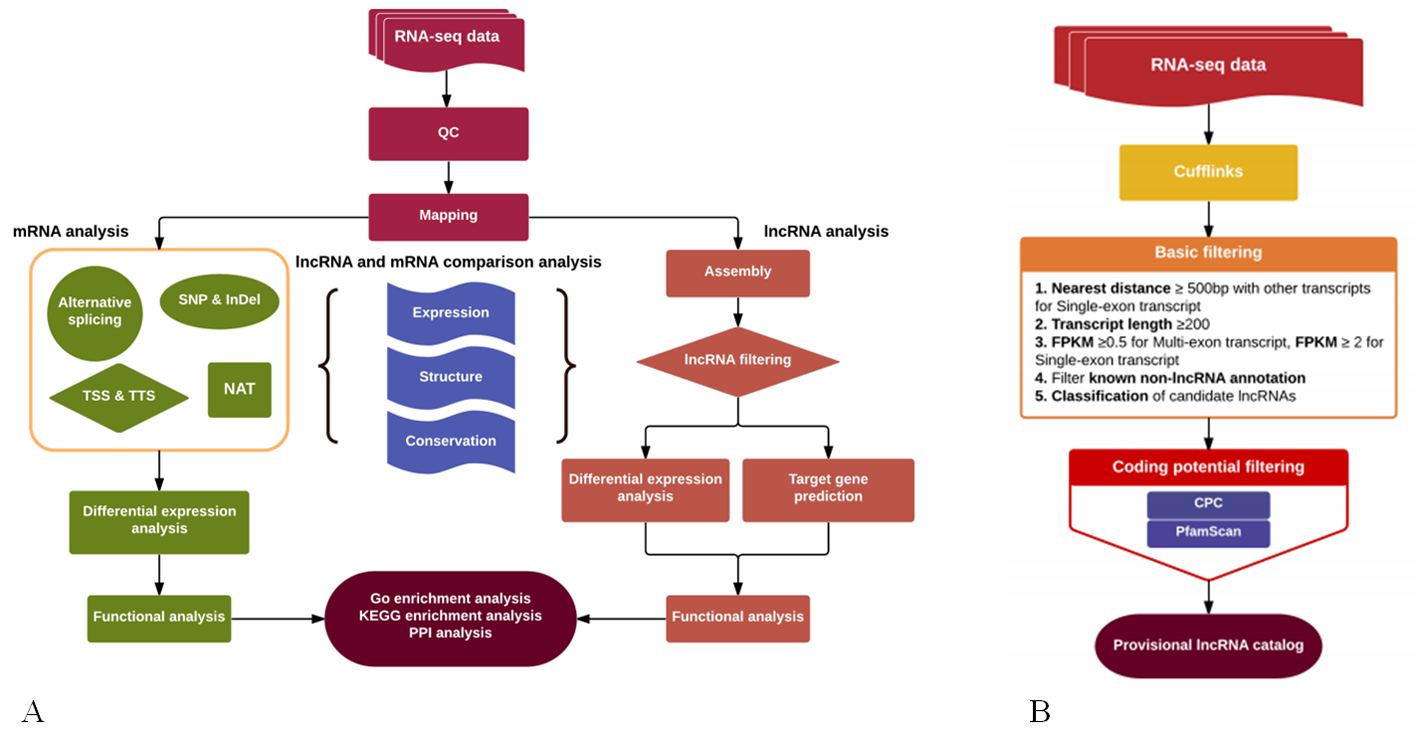

Supplement: S2 Fig — (TIF) [file pone.0200002.s002.tif]

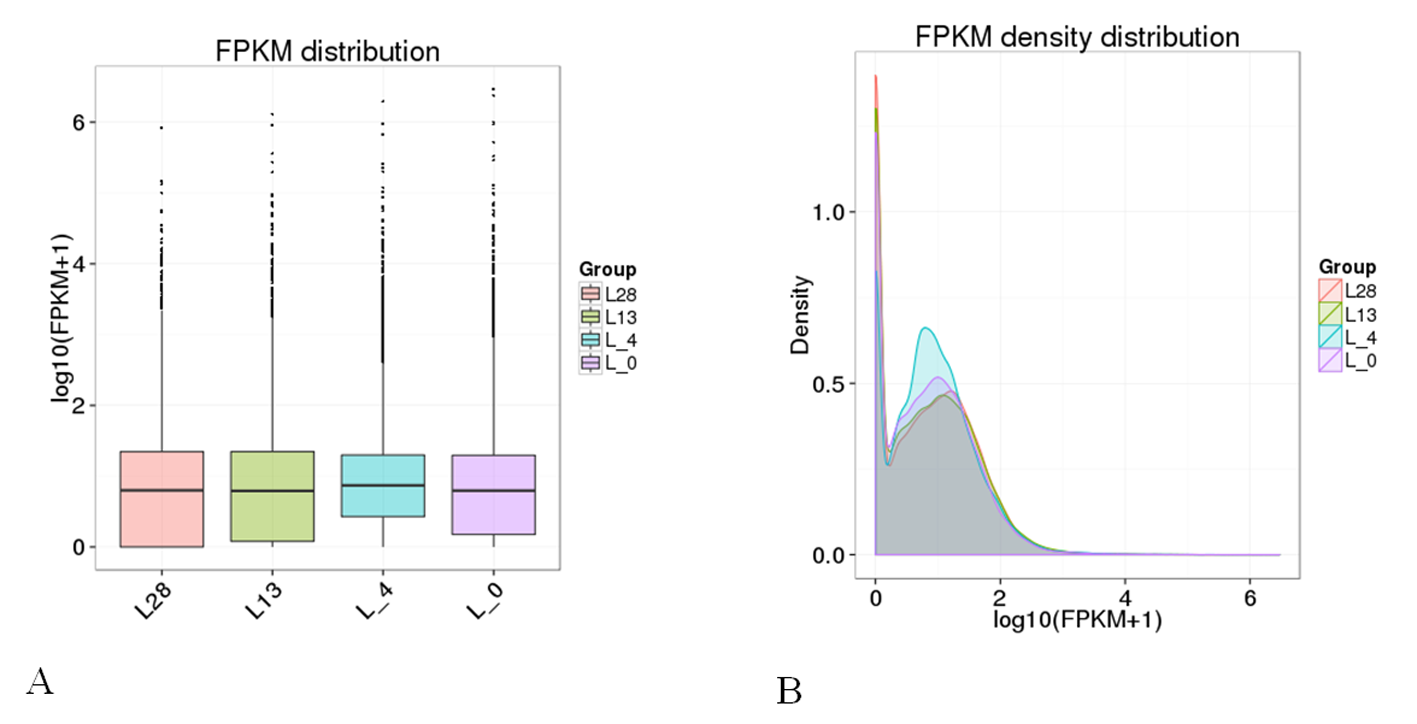

Supplement: S3 Fig — (TIF) [file pone.0200002.s003.tif]

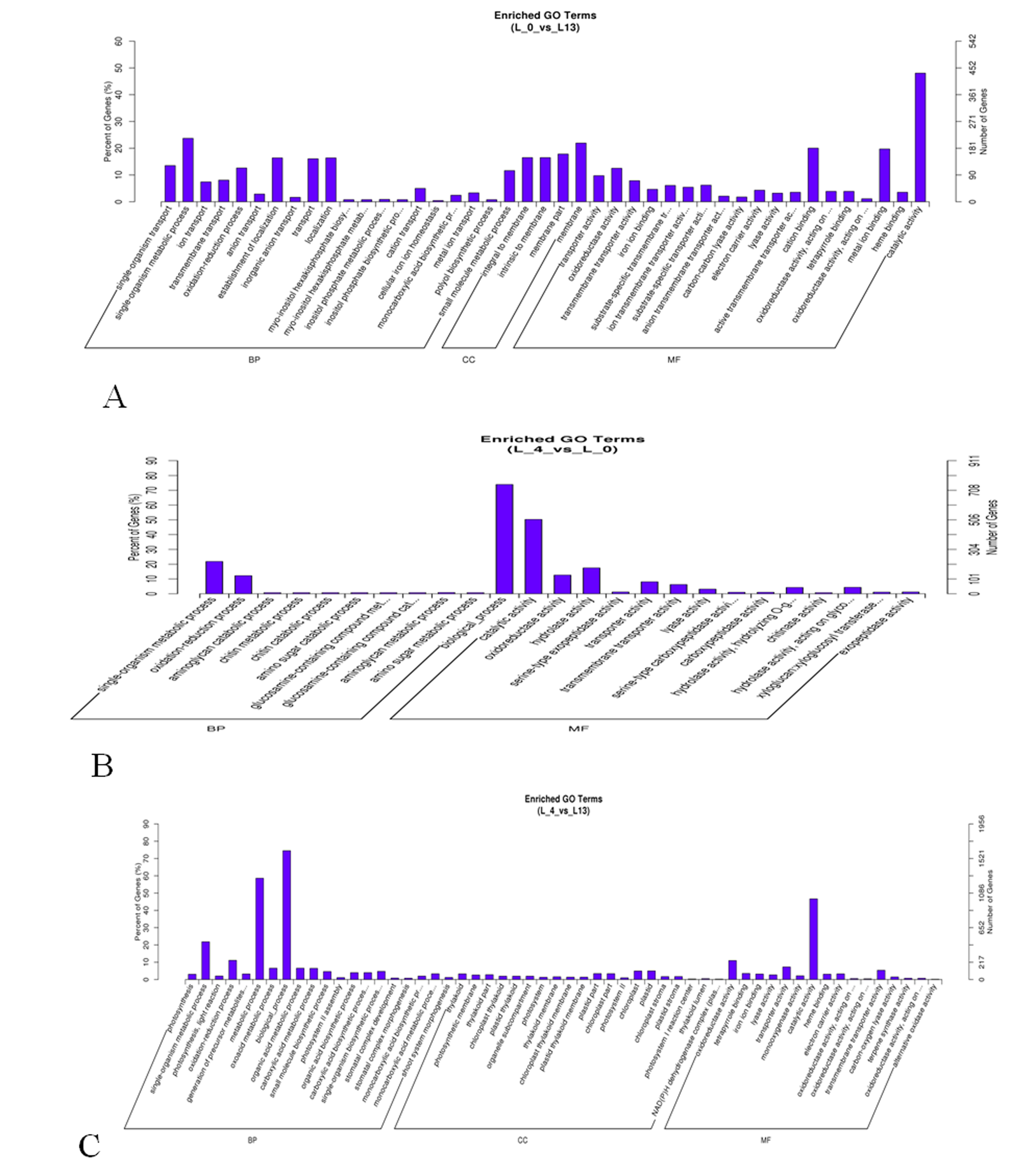

Supplement: S4 Fig — (TIF) [file pone.0200002.s004.tif]

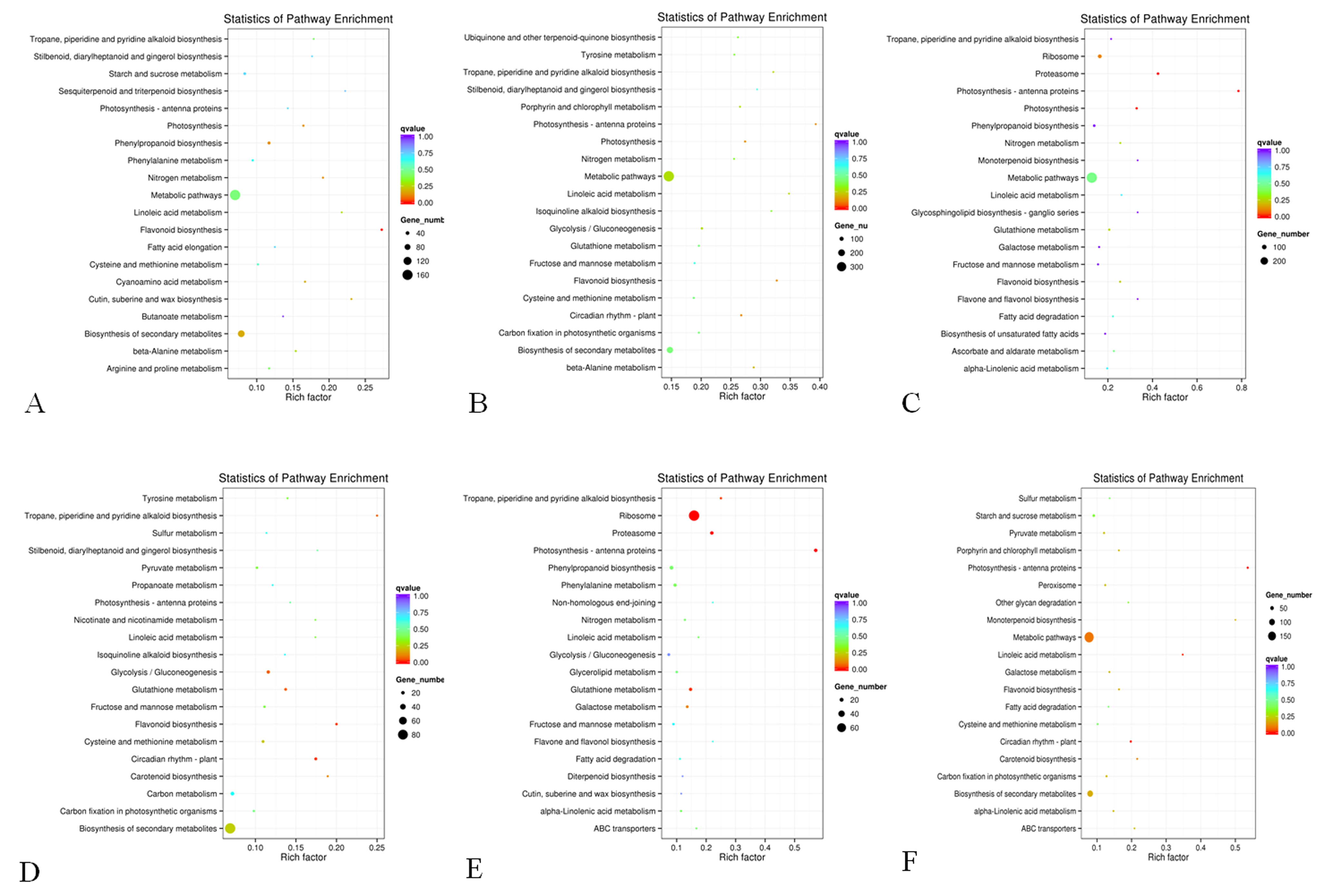

Supplement: S5 Fig — (TIF) [file pone.0200002.s005.tif]

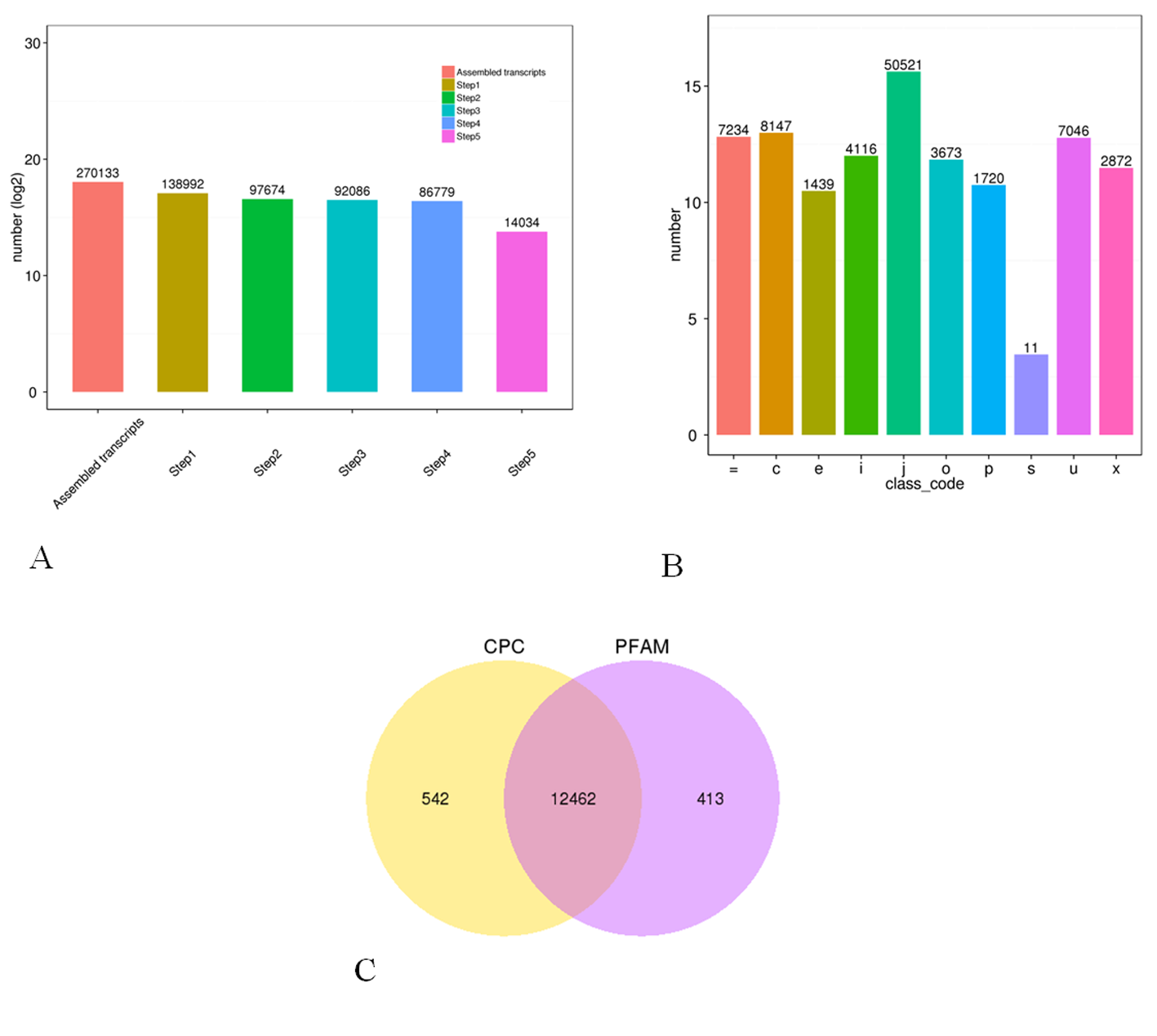

Supplement: S6 Fig — (TIF) [file pone.0200002.s006.tif]

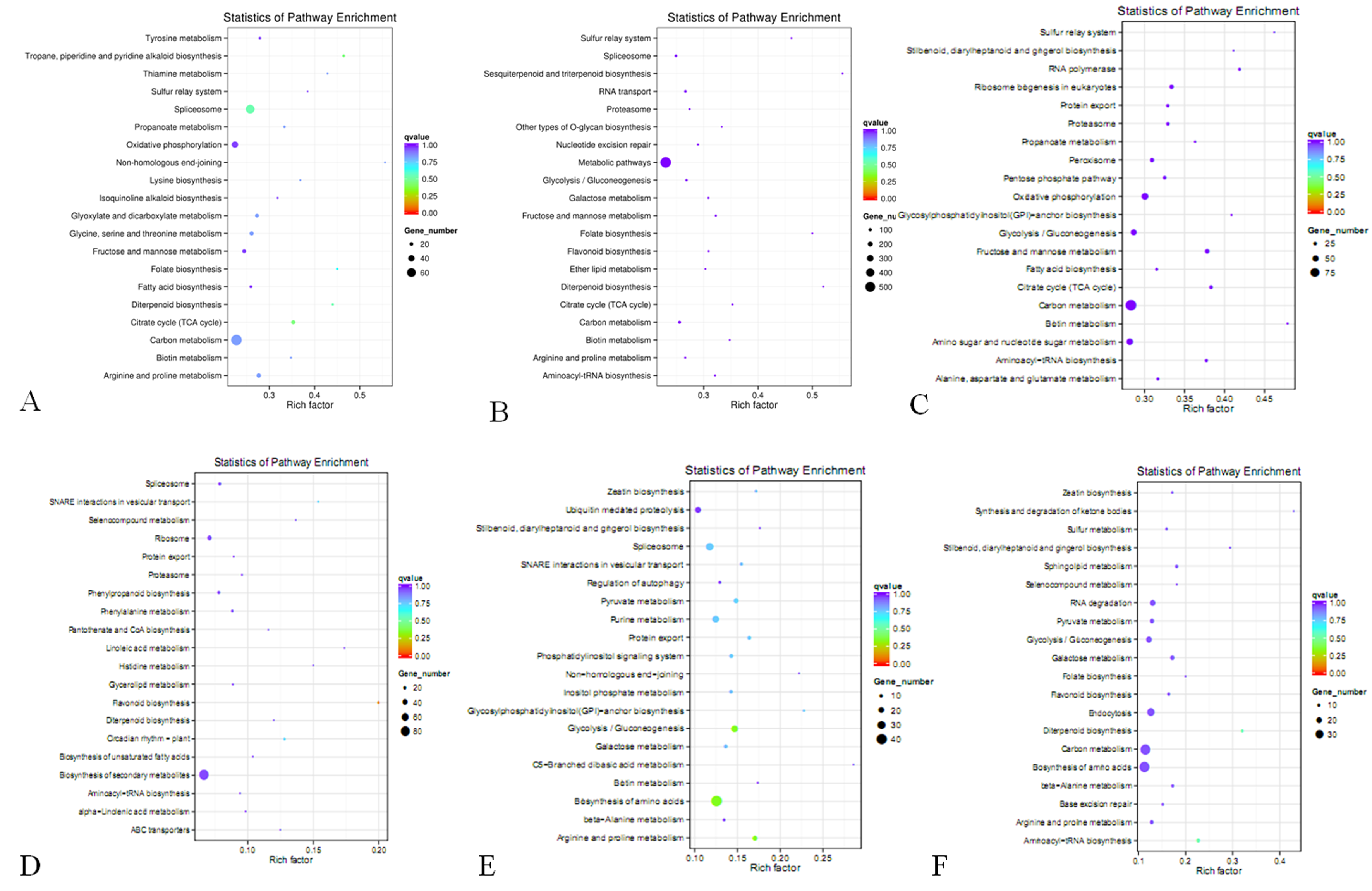

Supplement: S7 Fig — (TIF) [file pone.0200002.s007.tif]

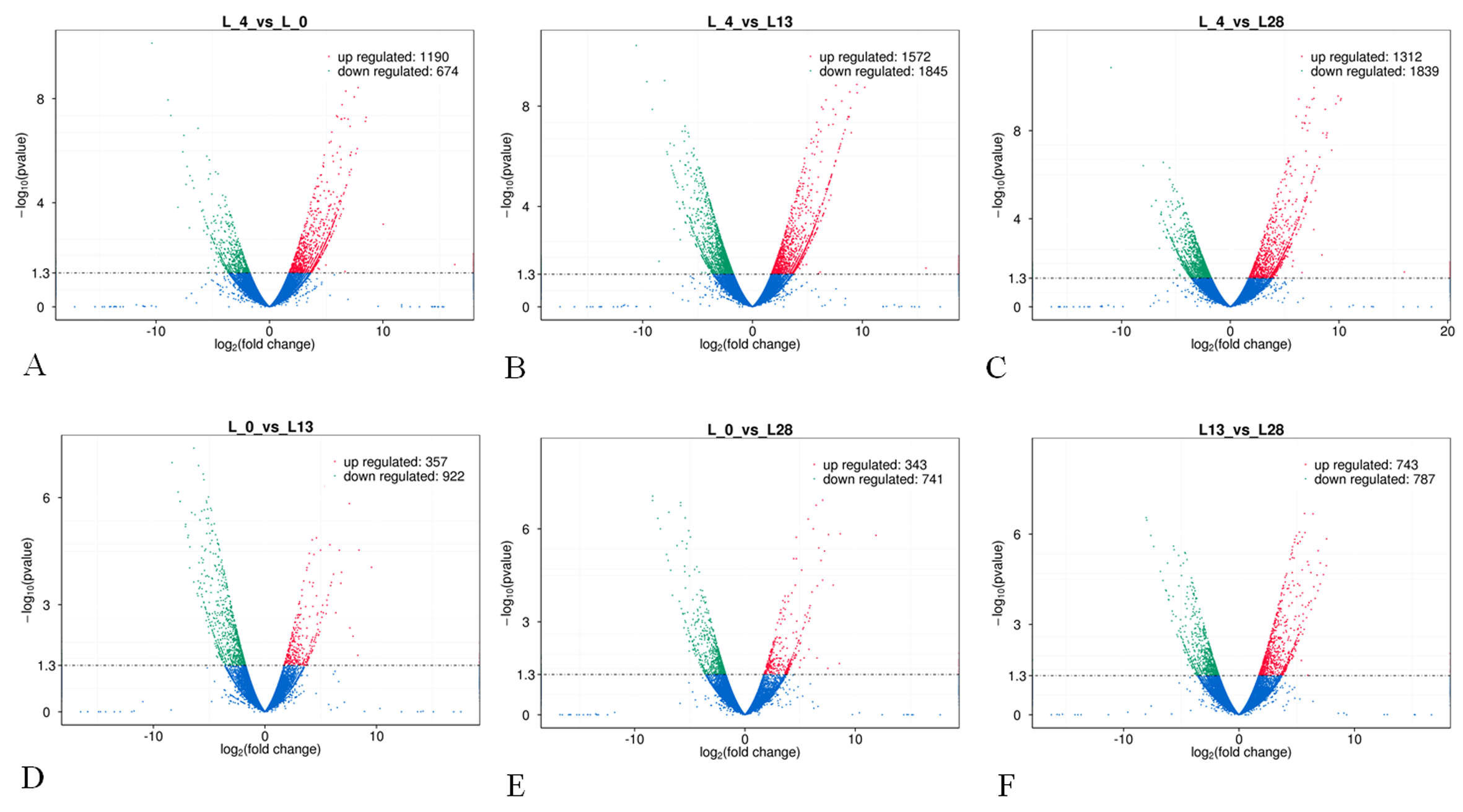

Supplement: S8 Fig — (TIF) [file pone.0200002.s008.tif]

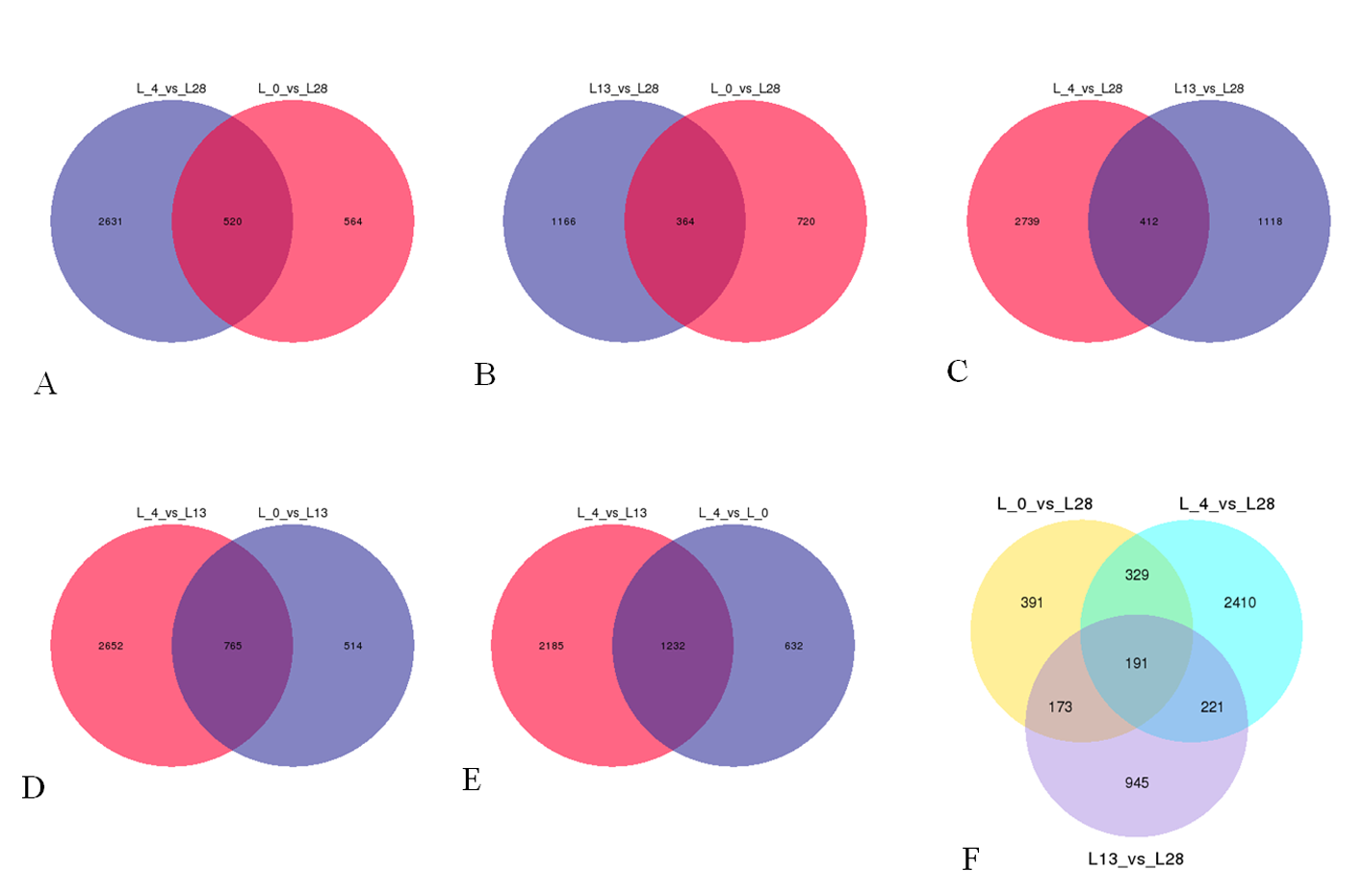

Supplement: S9 Fig — (TIF) [file pone.0200002.s009.tif]

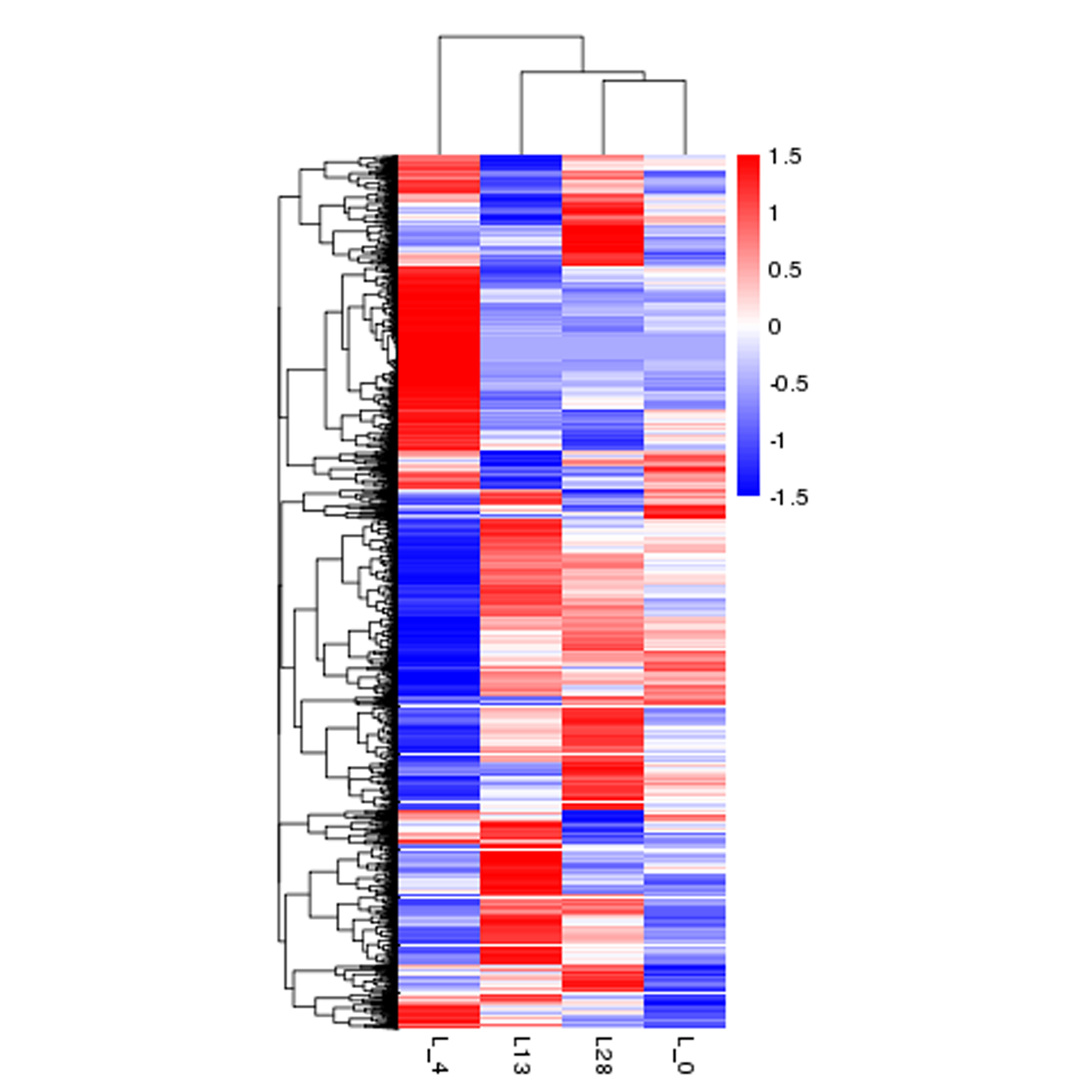

Supplement: S10 Fig — (TIF) [file pone.0200002.s010.tif]

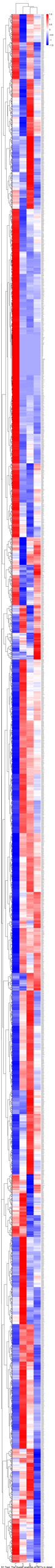

S1 Text. The cluster analysis of DETs in detail.

Supplement: S1 File — (PDF) [file pone.0200002.s030.pdf]
